# Adipose Tissue Is a Neglected Viral Reservoir and an Inflammatory Site during Chronic HIV and SIV Infection

**DOI:** 10.1371/journal.ppat.1005153

**Published:** 2015-09-24

**Authors:** Abderaouf Damouche, Thierry Lazure, Véronique Avettand-Fènoël, Nicolas Huot, Nathalie Dejucq-Rainsford, Anne-Pascale Satie, Adeline Mélard, Ludivine David, Céline Gommet, Jade Ghosn, Nicolas Noel, Guillaume Pourcher, Valérie Martinez, Stéphane Benoist, Véronique Béréziat, Antonio Cosma, Benoit Favier, Bruno Vaslin, Christine Rouzioux, Jacqueline Capeau, Michaela Müller-Trutwin, Nathalie Dereuddre-Bosquet, Roger Le Grand, Olivier Lambotte, Christine Bourgeois

**Affiliations:** 1 Université Paris Sud, UMR 1184, Le Kremlin-Bicêtre, France; 2 CEA, DSV/iMETI, IDMIT, Fontenay-aux-Roses, France; 3 INSERM, U1184, Immunology of Viral Infections and Autoimmune Diseases, Le Kremlin-Bicêtre, France; 4 Assistance Publique-Hôpitaux de Paris, Hôpital Bicêtre, Service d’anatomo-pathologie, Le Kremlin-Bicêtre, France; 5 Université Paris Descartes, Sorbonne Paris Cité, Faculté de Médecine, EA 7327, Paris, France; 6 Assistance Publique—Hôpitaux de Paris, Hôpital Necker-Enfants Malades, Laboratoire de Virologie, Paris, France; 7 Institut Pasteur, Unité HIV, Inflammation et Persistance, Paris, France; 8 INSERM, U1085-IRSET, Université de Rennes 1, Campus de Beaulieu, Rennes, France; 9 Assistance Publique—Hôpitaux de Paris, Hôpital Bicêtre, Service de Médecine Interne et Immunologie clinique, Le Kremlin-Bicêtre, France; 10 Assistance Publique—Hôpitaux de Paris, Hôpital Béclère, Service de Chirurgie Viscérale Minimale invasive, Clamart, France; 11 INSERM U972, Hôpital Paul Brousse, Villejuif, France; 12 Assistance Publique—Hôpitaux de Paris, Hôpital Antoine Béclère, Service de Médecine Interne et Immunologie clinique, Clamart, France; 13 Assistance Publique—Hôpitaux de Paris, Hôpital Bicêtre, Service de Chirurgie générale et digestive, Le Kremlin-Bicêtre, France; 14 INSERM UMR S938, CDR Saint-Antoine; Sorbonne Universités, UPMC Univ Paris 6, Paris, France; 15 Assistance Publique—Hôpitaux de Paris, Hôpital Tenon, Service de Biochimie et Hormonologie; ICAN, Institute of Cardiometabolism and Nutrition, Paris, France; Emory University, UNITED STATES

## Abstract

Two of the crucial aspects of human immunodeficiency virus (HIV) infection are (i) viral persistence in reservoirs (precluding viral eradication) and (ii) chronic inflammation (directly associated with all-cause morbidities in antiretroviral therapy (ART)-controlled HIV-infected patients). The objective of the present study was to assess the potential involvement of adipose tissue in these two aspects. Adipose tissue is composed of adipocytes and the stromal vascular fraction (SVF); the latter comprises immune cells such as CD4^+^ T cells and macrophages (both of which are important target cells for HIV). The inflammatory potential of adipose tissue has been extensively described in the context of obesity. During HIV infection, the inflammatory profile of adipose tissue has been revealed by the occurrence of lipodystrophies (primarily related to ART). Data on the impact of HIV on the SVF (especially in individuals not receiving ART) are scarce. We first analyzed the impact of simian immunodeficiency virus (SIV) infection on abdominal subcutaneous and visceral adipose tissues in SIVmac251 infected macaques and found that both adipocytes and adipose tissue immune cells were affected. The adipocyte density was elevated, and adipose tissue immune cells presented enhanced immune activation and/or inflammatory profiles. We detected cell-associated SIV DNA and RNA in the SVF and in sorted CD4^+^ T cells and macrophages from adipose tissue. We demonstrated that SVF cells (including CD4^+^ T cells) are infected in ART-controlled HIV-infected patients. Importantly, the production of HIV RNA was detected by *in situ* hybridization, and after the *in vitro* reactivation of sorted CD4^+^ T cells from adipose tissue. We thus identified adipose tissue as a crucial cofactor in both viral persistence and chronic immune activation/inflammation during HIV infection. These observations open up new therapeutic strategies for limiting the size of the viral reservoir and decreasing low-grade chronic inflammation via the modulation of adipose tissue-related pathways.

## Author Summary

Chronic immune activation/inflammation and viral persistence in reservoirs are important features of chronic HIV infection—even in patients receiving ART. We sought to evaluate the involvement of adipose tissue in chronic HIV/SIV infections. Adipose tissue accounts for 15 to 20% of the body weight, contains both adipocytes and (within the stromal vascular fraction) immune cells, and exerts crucial metabolic and immune activities. We postulated that adipose tissue might provide an ideal environment for HIV persistence and immune inflammation. We first showed that viremic SIV-infected macaques had elevated levels of immune activation and inflammation in adipose tissue, and that both resident CD4^+^ T cells and macrophages were infected. In similar experiments in ART-controlled HIV-infected patients, HIV DNA was detected in the stromal vascular fraction of adipose tissue (more specifically, in adipose tissue CD4^+^ T cells). Replication-competent HIV was detected in *ex vivo-* activated, sorted adipose tissue CD4^+^ T cells from six aviremic, ART-treated patients. Thus, adipose tissue may constitute a viral reservoir and be involved in long-term immune activation and inflammation—even in ART-suppressed patients. Given that adipose tissue is strongly regulated by both metabolic and immune pathways, modulating adipose tissue may constitute a valuable means of limiting both viral persistence and chronic inflammation in HIV-infected patients even ART-controlled.

## Introduction

Human immunodeficiency virus (HIV) infection is characterized by massive CD4^+^ T cell depletion in the intestinal mucosa (progressively affecting blood and lymphoid CD4^+^ T cells) and sustained systemic immune activation and inflammation. The advent of antiretroviral therapy (ART) has drastically changed the outcomes of HIV infection by enabling a reduction in the viral load and the restoration (at least in part) of CD4^+^ T cell counts. In people receiving ART, chronic HIV infection is characterized by the persistence of viral reservoirs (preventing the eradication of HIV) and chronic immune activation and inflammation (which are associated with all-cause, non-AIDS-related morbidity, such as cardiovascular disease and non-insulin dependent diabetes, and mortality [1–3]. Similar observations (i.e. viral persistence and low level immune activation and inflammation) apply–albeit to a lesser extent—to “HIV-controllers”, i.e. patients who are able to spontaneously control viral load [4,5]. The eradication or reduction of viral reservoirs remains a crucial therapeutic objective in the fight against HIV [6], and both cellular and anatomical reservoirs require further investigation [7,8]. A second therapeutic objective is to circumscribe the sustained immune activation. It has been suggested that microbial translocation is a potent factor in the maintenance of chronic immune activation/inflammation [9], along with viral persistence, CD4^+^ T cell lymphopenia, Th17 loss, a change in the regulatory T cell balance, disruption of the lymph node architecture, viral co-infection, accelerated ageing, the side effects of some antiretroviral drugs, and individual susceptibility [2,10,11]). HIV-infected patients on ART are not always able to reestablish gut mucosa integrity and/or normal CD4^+^ T cell counts, and chronic, low-levels immune activation appears to persist [12]. Taken as a whole, these data suggest that (i) immune activation and chronic inflammation are driven by multiple factors and (ii) targeting several inflammatory mechanisms may achieve better immune restoration.

We hypothesized that adipose tissue has an important role in both chronic immune activation/inflammation and viral persistence. In fact, adipose tissue is not merely a metabolic and endocrine organ for lipid storage; it also exhibits strong immune activity: (a) adipose tissue is an important site of production of both pro-inflammatory molecules (such as leptin, IL-6, MCP-1 (CCL2), RANTES (CCL5) and TNF-α) and anti-inflammatory adipokines (such as adiponectin) [13–21]; (b) the stromal vascular fraction (SVF) contains immune cells (CD4^+^ T cells and macrophages) that are potentially important target cells for HIV; and (c) a growing body of evidence demonstrates the close relationship between the immune response and metabolic alterations [22,23], such as the recruitment of activated CD8^+^ T cells and inflammatory macrophages and their participation in inflammation processes (and then the modifications of the adipose tissue) in obesity and non-insulin dependent diabetes [24–31]. In the context of obesity, the interplay between adipocytes and immune cells is being actively investigated. The two cell types are clearly “partners in inflammation” as their coordinated action leads to adipose inflammation [32]. Studies of the adipose tissue during HIV infection have essentially addressed the toxicity of certain antiretroviral drugs and their induction of metabolic alterations–even though a direct impact of infection *per se* was clearly documented by early studies [33,34]. Metabolic alterations [34–38] and elevated levels of pro-inflammatory cytokines have been described in both plasma and adipose tissue [39–43]. Previous analyses of adipocytes failed to demonstrate that adipocytes could be infected by HIV *in vivo* [44]—in contrast to the results of *in vitro* studies [45–47]. However, it has been shown that the HIV viral proteins Vpr and Nef are present in adipose tissue and have a negative impact on adipose homeostasis [48–50]. These observations provide a strong rationale for reconsidering the impact of HIV infection on adipose tissue by focusing on immune cells rather than adipocytes. We hypothesize that adipose tissue may constitute a neglected partner that drives viral persistence and chronic immune activation via two nonexclusive mechanisms: local infection and the abnormal local activation of immune cells. To assess the putative infection of adipose immune cells more precisely, we first analyzed tissues from chronically SIV-infected macaques. We chose to analyze both subcutaneous adipose tissue (SCAT) and visceral adipose tissue (VAT) because they differ in terms of metabolic activity and immune cell content [14,15,17,51]. Secondly, we extended these analyses to ART-treated HIV-infected patients.

In the present report, we demonstrate that SIV infection is associated with changes in the composition of adipose tissue, such as elevated densities of both adipocytes and stromal vascular cells. Importantly, adipose tissue macrophages and CD4^+^ and CD8^+^ T cells exhibited a more intense activation profile (relative to non-infected animals). Furthermore, SIV DNA and RNA was detected in total SVF and in sorted adipose tissue macrophages and CD4^+^ T cells. We observed similar results in ART-controlled, HIV-infected patients having undergone elective visceral surgery: their SVF samples were positive for HIV DNA. The presence of infected/virus-producing cells within adipose tissue was confirmed by the detection of HIV RNA in tissue sections via *in situ* hybridization. Lastly, we performed an *in vitro* reactivation assay on samples from six patients and found that adipose tissue CD4^+^ T cells were capable of producing replication-competent virus. Taken as a whole, our data show that adipose tissue as a viral reservoir with inflammatory potential.

## Results

### SIV infection influences the density of adipocytes and stromal vascular cells

**Fig 1:**
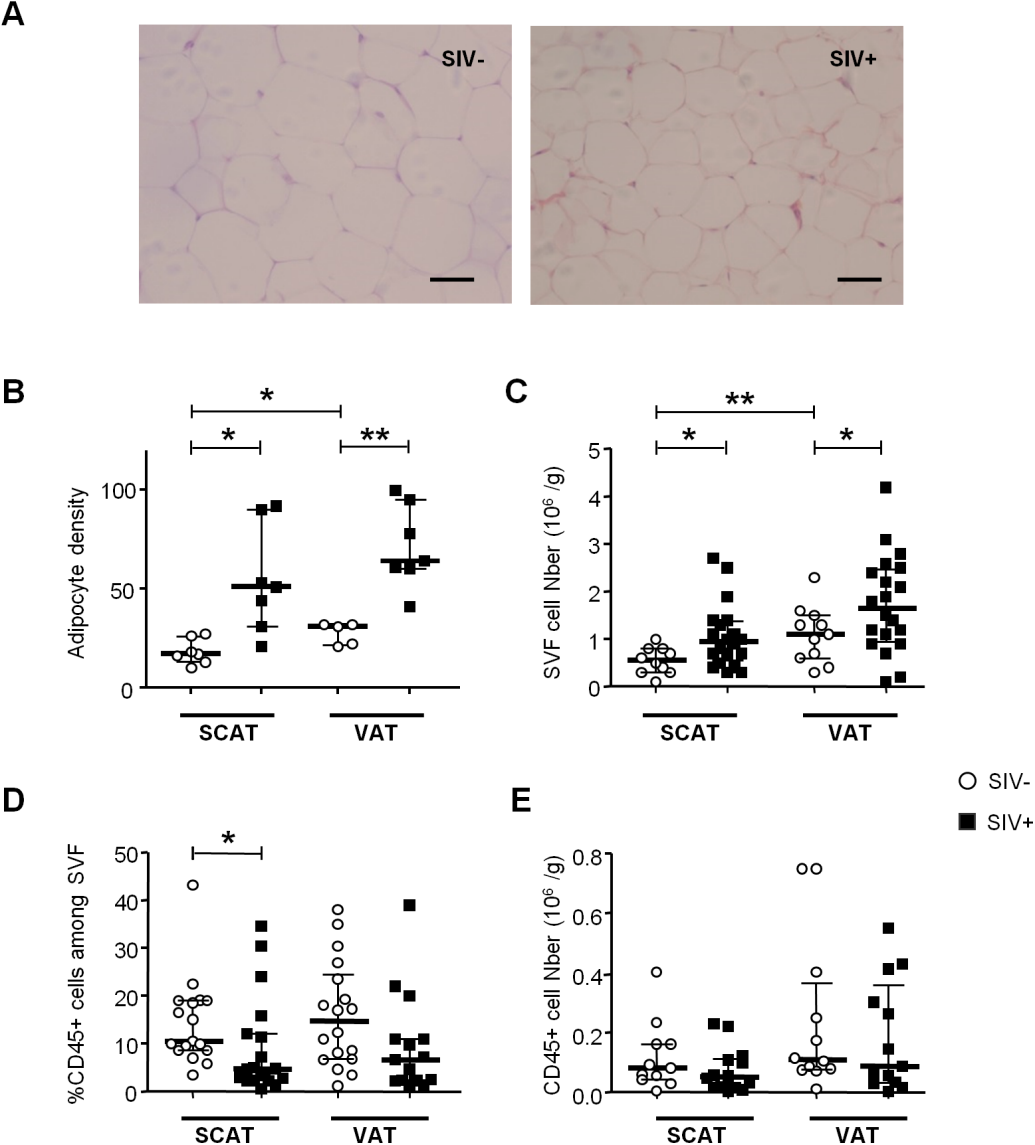
The influence of SIV infection on adipocyte and SVF cell density. (A) Representative adipose tissue sections of SIV-infected and non-infected macaques. Scale bar: 50μm, magnification x 250. (B) The adipocyte density (numbers per HPF) in SCAT and VAT from 5 to 7 non-infected animals (SIV-) and 7 SIV-infected animals (SIV+). (C) Adipose tissue dissociation and counting of cells in the SVF was performed with 20 SIV+ animals and 10 SIV- animals. Counts are expressed per gram of adipose tissue. (D, E) Flow cytometry of the recovered SVF, yielding the proportion (D) and number (E) of CD45-expressing cells per gram of tissue. The analysis was performed for both SCAT and VAT; SIV- animals (n = 10) are represented by open circles and SIV+ animals (n = 12) are represented by filled squares. Data are quoted as the median [interquartile range]. Significant differences in a Mann-Whitney non-parametric test are shown as * p<0.05; ** p<0.01.

We first determined the impact of SIV infection on adipocyte density (as evaluated by microscopy) (Fig 1A). Adipocyte density in both SCAT and VAT was markedly higher in SIV-infected macaques than in non-infected animals (median [interquartile range] number of adipocytes per field in SCAT: 51 [31–90] in infected animals and 17 [13–26] in controls, p = 0.032; in VAT: 64 [60–95] in infected animals and 31 [21–32] in controls, p = 0.0059) (Fig 1B). We also counted SVF cells harvested from SCAT and VAT in infected and non-infected animals (Fig 1C). To enable a valid comparison, SVF cell counts were expressed per gram of adipose tissue. In line with previous publications [47,52], we found that non-infected animals had significantly higher numbers of SVF cells in VAT than in SCAT (p = 0.009). Significantly higher SVF cell counts were detected in SCAT and VAT from infected animals than in non-infected animals (p<0.05 for both tissues).

We next determined the proportion of CD45-expressing cells in SVF. CD45^+^ cells accounted for a small proportion of SVF cells in both SCAT and VAT recovered from non-infected animals. In animals with chronic SIV infection, we observed significantly lower percentages of CD45^+^ cells in SCAT SVF (relative to non-infected animals) (Fig 1D). A similar trend was detected in VAT. To determine whether these lower percentages of CD45^+^ cells reflected a fall in CD45^+^ cell numbers or the recruitment/expansion of CD45^-^ cells, we analyzed the absolute CD45^+^ cell count. As shown in Fig 1E, groups of infected and non-infected animals did not differ significantly in terms of the number of CD45^+^ cells recovered from SCAT or VAT; this was suggestive of changes in the numbers of CD45^-^ cells. We thus demonstrated that chronic SIV infection modulated both adipocyte and SVF cells. In the latter cell population, the quantitative alteration essentially concerned CD45^-^ cells.

### SIV infection influences the characteristics of adipose tissue T lymphocytes

**Fig 2:**
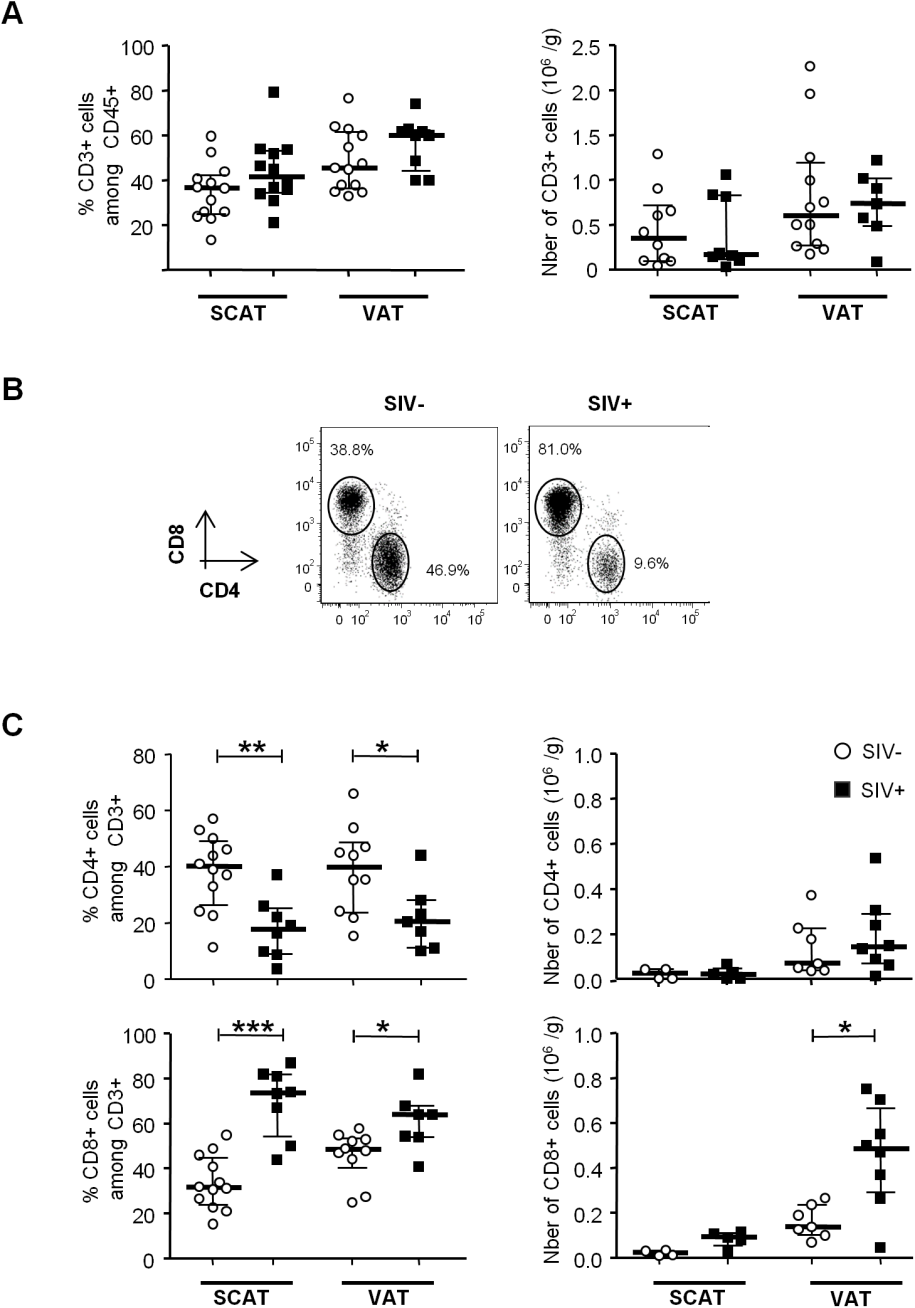
The influence of SIV infection on adipose-resident T cell subsets. (A) Percentages and numbers (expressed per gram of adipose tissue recovered) of CD3-expressing cells among the CD45^+^ fraction from SCAT and VAT in non-infected animals (open circles) and SIV-infected animals (filled squares). Percentages were derived for 12 to 13 animals per group and counts were derived for 8 to 12 animals. (B) Representative dot plots showing CD4 and CD8 expression among CD3^+^ T cells in SIV-infected and non-infected animals. (C) Percentages and numbers (expressed per gram of adipose tissue) of CD4- and CD8-expressing cells among CD3^+^ cells recovered from SCAT and VAT of non-infected animals (open circles) and SIV-infected animals (filled squares). Percentages were derived for 8 to 12 animals per group and counts were derived for 5 to 8 animals. Data are quoted as the median [interquartile range]. Significant differences in a Mann-Whitney non-parametric test are shown as * p<0.05; ** p<0.01; *** p<0.001.

We next looked at whether or not SIV infection was associated with changes in the percentages of CD4^+^ and CD8^+^ T lymphocytes within adipose tissue. To this end, we determined the percentage of total T lymphocytes among CD45-expressing cells and the percentages of CD4^+^ and CD8^+^ T lymphocytes among CD3-expressing cells (Fig 2). Adipose tissue T lymphocytes accounted for approximately half of the CD45^+^ cells within the SVF; SIV-infected and non-infected animals did not differ significantly in terms of the proportion and number of CD3^+^ cells (Fig 2A).

**Fig 3:**
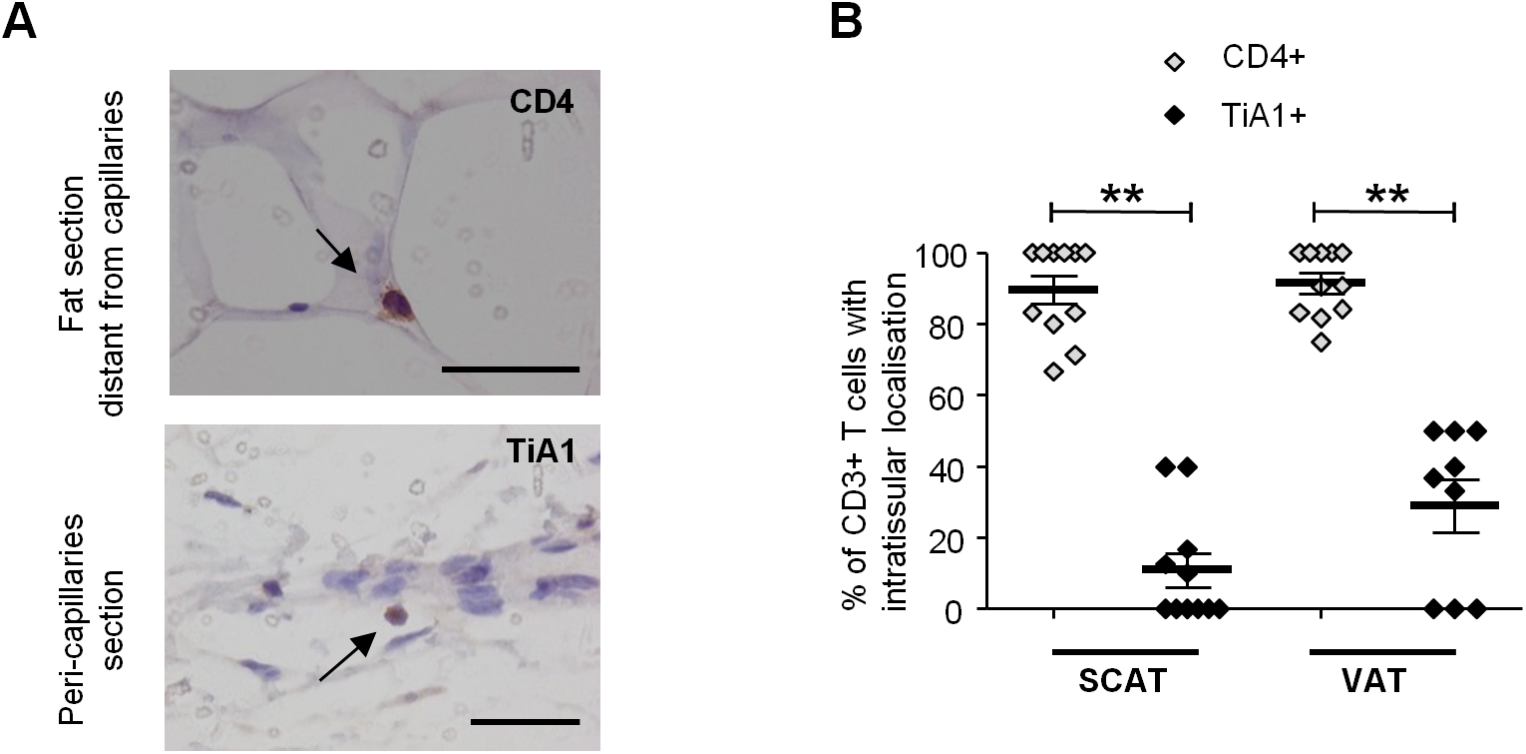
Localization of T cells in the adipose tissue of SIV-infected animals. Immunochemical analyses were performed on 11 SCAT and 10 VAT of SIV-infected animals. (A) Representative tissue sections showing CD4^+^ T cells within fat and far from capillaries (upper) and TiA1^+^ cells in the vicinity of the capillaries (lower). Scale bar: 50 μm, magnification x640 for CD4, x500 for TiA1. (B) Percentages of CD4^+^ T cells (open diamonds) and CD8^+^ T cells (filled diamonds) far from capillaries, corresponding to adipose-resident lymphocyte subsets. Depending on the location, cells were counted after immunochemical staining on SCAT and VAT sections from SIV-infected animals. Data are quoted as the median [interquartile range]. Significant differences in a Mann-Whitney non-parametric test are shown as ** p<0.01.

However, SIV infection was associated with differences in CD4^+^ and CD8^+^ T cell relative percentages (Fig 2B and 2C). The proportion of CD4^+^ T cells was significantly lower in SIV-infected macaques (17.6% [8.9–25] in SCAT, and 20.4% [11.0–28.0] in VAT) than in non-infected animals (40.0% [26.4–49.0] in SCAT, and 39.8% [23.6–48.7] in VAT; p = 0.0043 for SCAT, p = 0.0317 for VAT). Conversely, CD8^+^ T cell percentages were significantly higher in infected animals. The low CD4^+^ T cell percentages may reflect the CD4^+^ T cell depletion associated with SIV infection, whereas elevated CD8^+^ T cell percentages may reflect either a passive increase (due to CD4^+^ T cell decay), an active increase due to antiviral CD8 recruitment (as described in lungs after SIV infection [53,54]), or viral-independent local recruitment (as described in inflammatory adiposity [27]). To evaluate the direct impact of SIV infection on T cell subsets, we analyzed the numbers of CD4^+^ and CD8^+^ T cells recovered from adipose tissue of infected and non-infected groups of animals. Surprisingly, the CD4^+^ T cell number was not lower in infected animals, and there was even a non-significant trend towards CD4^+^ T cell accumulation in VAT (Fig 2C). In contrast, CD8^+^ T cell numbers in VAT were significantly higher (0.48 10^6^ [0.29–0.67] in SIV-infected animals than in non-infected animals (0.14 10^6^ cells/g [0.11–0.24]; p = 0.0205). A similar trend was observed for SCAT. Thus, the change in the CD4/CD8 ratio was mainly driven by an increase in CD8^+^ T cell numbers—a phenomenon that is often associated with inflammatory adiposity [27]. Lastly, we used immunochemical techniques to formally confirm the presence of T lymphocytes in adipose tissue (Fig 3A). Given that the recruitment of peripheral blood cells into adipose tissue during local inflammation has been described, we evaluated the T cell distribution both in the vicinity of the capillaries and far from the capillaries in SIV-infected animals. The CD4^+^ T cells were mainly located far from the capillaries, whereas CD8^+^ T cells were essentially located in the capillary area (Fig 3B). These observations are in accordance with massive influx of CD8^+^ T cells previously described in the context of adipose inflammation [27]. In contrast, CD4^+^ T cell counts in adipose tissue were only slightly affected by SIV infection, and most CD4^+^ T cells recovered in the SVF had not recently migrated into the adipose tissue from peripheral blood.

**Fig 4:**
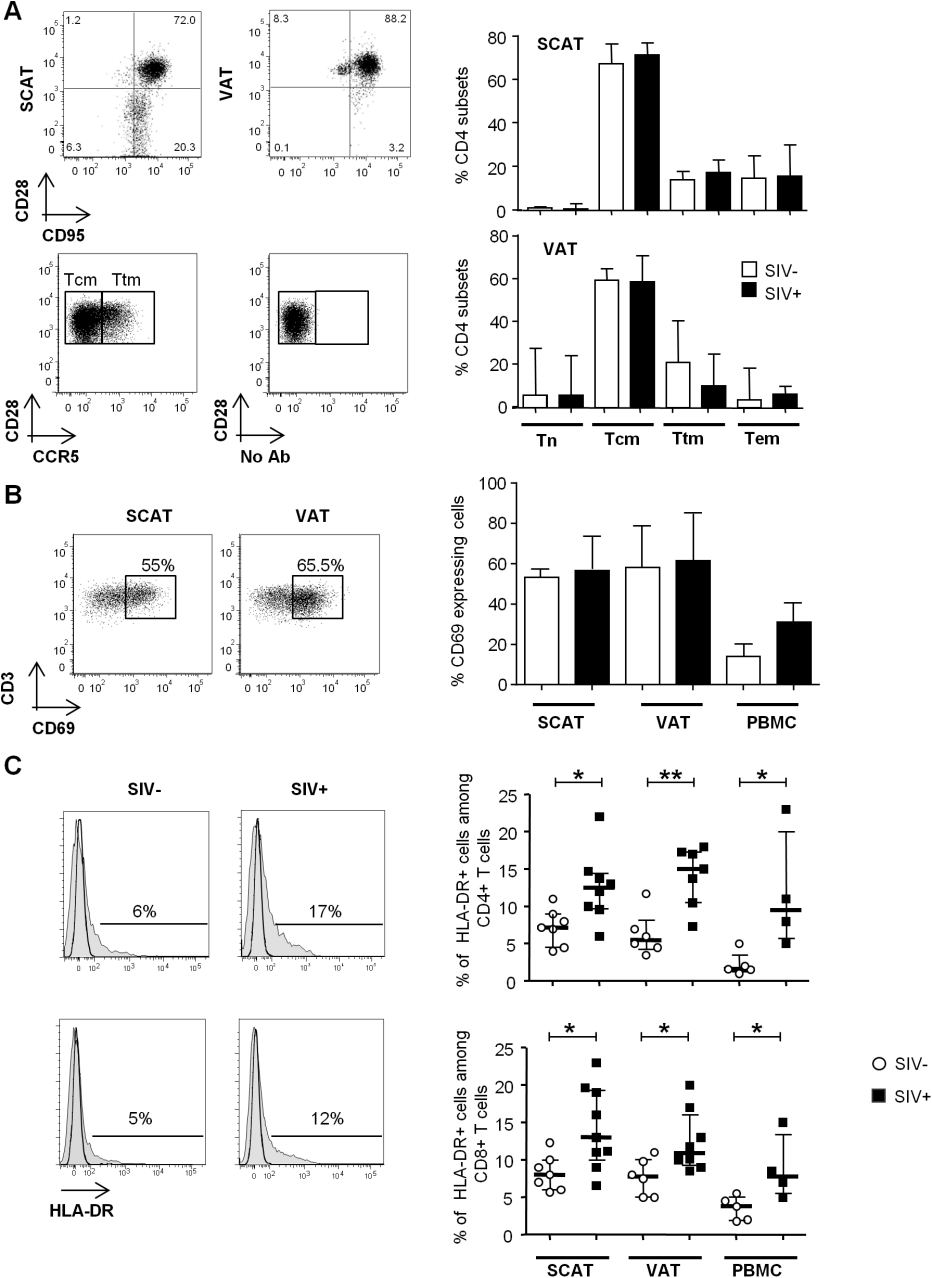
The influence of SIV infection on T cell differentiation and activation status. (A) The naïve and memory CD4^+^ T cell subset distribution in SCAT and VAT from SIV-infected or non-infected animals. Representative dot plots showing the gating strategies used to define Tn, Tcm, Ttm and Tem subsets among the CD4^+^ T cells, based on CD28, CD95 (upper dot plot, SCAT and VAT samples). CCR5 expression and the FMO profile in CD28+CD95+ fractions are shown (lower dot plot: VAT sample). The right-hand panels show the distribution of CD4^+^ T cells among the various subsets in non-infected animals (n = 5, open column) and SIV-infected animals (n = 7, filled column). (B) CD69 expression on CD4^+^ T cells recovered from SCAT, VAT and PBMCs from SIV-infected or non-infected animals. Representative dot plots for SCAT and VAT (left panel) and the percentages of CD69-expressing CD4^+^ T cells in SCAT, VAT and PBMCs from non-infected animals (n = 6, open columns) and SIV-infected animals (n = 7, filled columns) are shown. (C) HLA-DR expression on SCAT, VAT and PBMCs from SIV-infected or non-infected animals. Representative histograms showing HLA-DR expression on adipose-resident CD4^+^ and CD8^+^ T cells recovered from SCAT from non-infected (left panel) and SIV-infected animals (right-hand panel). HLA-DR expression histograms are shown in plain histogram. FMO staining (open histograms) was used to define the gating strategy. The percentage of HLA-DR-expressing cells among CD4^+^ and CD8^+^ T cells recovered from SCAT, VAT and PBMCs from non-infected animals (n = 6, open circles) and SIV-infected animals (n = 4–8, filled squares). Data are quoted as the median [interquartile range]. Significant differences in a Mann-Whitney non-parametric test are shown as * p<0.05; ** p<0.01.

We next evaluated the differentiation of adipose CD4^+^ and CD8^+^ T cells obtained from infected and non-infected animals (Figs 4 and S1). Testing for CD95, CD28 and CCR5 expression enabled us to identify naïve (Tn), central memory (Tcm), transitional memory (Ttm) and effector memory (Tem) subsets (Figs 4A and S1A) [55,56]. In both infected and non-infected animals, naïve CD4^+^ T cells (CD95- CD28int) were virtually absent, whereas the Tcm (CD95^+^ CD28^+^ CCR5^-^) fraction was predominant in both SCAT and VAT. Interestingly, this profile was specific to CD4^+^ T cells since CD8^+^ T cells from adipose tissue were essentially Tem (CD95^+^ CD28^-^) (S1 Fig). Among CD4^+^ T cells, the CD95^+^ CD28^+^ CD4^+^ T cell fraction which includes the two potential cellular CD4^+^ T cell reservoir, i.e. Tcm (CCR5^-^) and Ttm (CCR5^+^), accounted for 82.3% [69.0–89.0] of the total in SCAT and 77.6% [69.9–77.6] in VAT, vs. 45.3% [28.8–62.0] in peripheral blood mononuclear cells (PBMCs) (p = 0.001 and 0.014 respectively) (S2A and S2B Fig). Interestingly, we did not detect significant differences in any of the CD4^+^ T cell fractions when comparing adipose tissue from SIV-infected animals (n = 7) and non-infected animals (n = 5). To evaluate the proportion of resident memory T cells [57,58], we next determined CD69 expression on CD4^+^ T cells recovered from adipose tissue and (as a control) in PBMCs (Fig 4B). The fraction of CD4^+^ T cells expressing CD69 was significantly higher in adipose tissue than in PBMCs (p = 0.0003 for SCAT and 0.0034 for VAT). However, SIV infection was not associated with a significant difference in the proportion of CD69-expressing cells. Thus, the maintenance of normal CD4^+^ T cell numbers was associated with preservation of memory CD4^+^ T cell subset distribution in general as well as the resident CD4^+^ T cell memory distribution.

Lastly, we evaluated the activation profile of adipose tissue T cells during chronic SIV infection (Fig 4C). We determined the expression of HLA-DR (a standard marker of T cell activation) on adipose tissue CD4^+^ and CD8^+^ T cells and (as a control) in PBMCs. In non-infected animals, there were no significant differences between SCAT and VAT in terms of CD4^+^ and CD8^+^ T cell activation (CD4^+^ T cells: 7.2% [4.5–9] in SCAT and 5.5% [4.3–8.2] in VAT; CD8^+^ T cells: 8.0% [6.0–10.0] in SCAT and 7.8% [5.0–10.1] in VAT). The percentages of HLA-DR-expressing T cells were higher in both SCAT and VAT than for PBMCs (1.6% [1.3–3.5] for CD4^+^ T cells in PBMCs and 3.8 [1.9–5.0] for CD8^+^ T cells in PBMCs). In SIV-infected animals, the percentage of HLA-DR-expressing cells was significantly higher in CD4^+^ and CD8^+^ T cells recovered in SCAT, VAT and PBMCs, relative to non-infected controls (Fig 4C). For example, the percentage of HLA-DR-expressing CD4^+^ T cells was 12.5% [5.5–15] in SCAT, 15.0% [10.5–17.3] in VAT and 9.5% [5.8–20.0] in PBMCs from SIV-infected animals. Interestingly, we could not detect any significant difference in the proportion of Ki-67-expressing CD4^+^ or CD8^+^ T cells (S3 Fig), suggesting that change in activation profile was not associated with massive *in situ* proliferation. Overall, SIV infection slightly affected CD4^+^ T cell numbers and their differentiation profile, and was associated with increased T cell activation in adipose tissue.

A constant concern when studying immunity in tissues is the potential bias induced by blood contamination. Here, special efforts were made to avoid this; adipose tissue was devascularized and washed in medium prior to digestion. Importantly, the low observed proportion of CD45-expressing cells constitutes important evidence of low blood contamination. It is noteworthy that B cells (identified as CD20-expressing cells) were virtually absent from SCAT (0.10% of SVF cells [0.01–0.40]) and VAT (0.11% [0.01–1.10]) but were detected in PBMCs (9.5% [3.0–10.0]) (S2C Fig). The absence of B cells is thus a reliable indicator of the absence of blood contamination of adipose tissue samples. Moreover, adipose tissue CD4^+^ T cells differed significantly from PBMCs, with significantly lower percentages of naïve cells (the CD4^+^ Tn fraction represented 2.0% [0.3–3.6] of the cells in SCAT, 5.6% [1.3–22.0] in VAT and 22.0% [8.9–41.7] in PBMCs; p<0.0001 and p = 0.03, respectively) and higher percentages of the two memory T cell subsets that are preferentially infected (i.e. Tcm and Ttm) (S2A and S2B Fig). Lastly, the immunochemical demonstration of different localizations of CD4^+^ and CD8^+^ T cells (Fig 3) also suggests that blood contamination was absent or barely present.

### SIV infection influences the phenotype of adipose-resident macrophages

**Fig 5:**
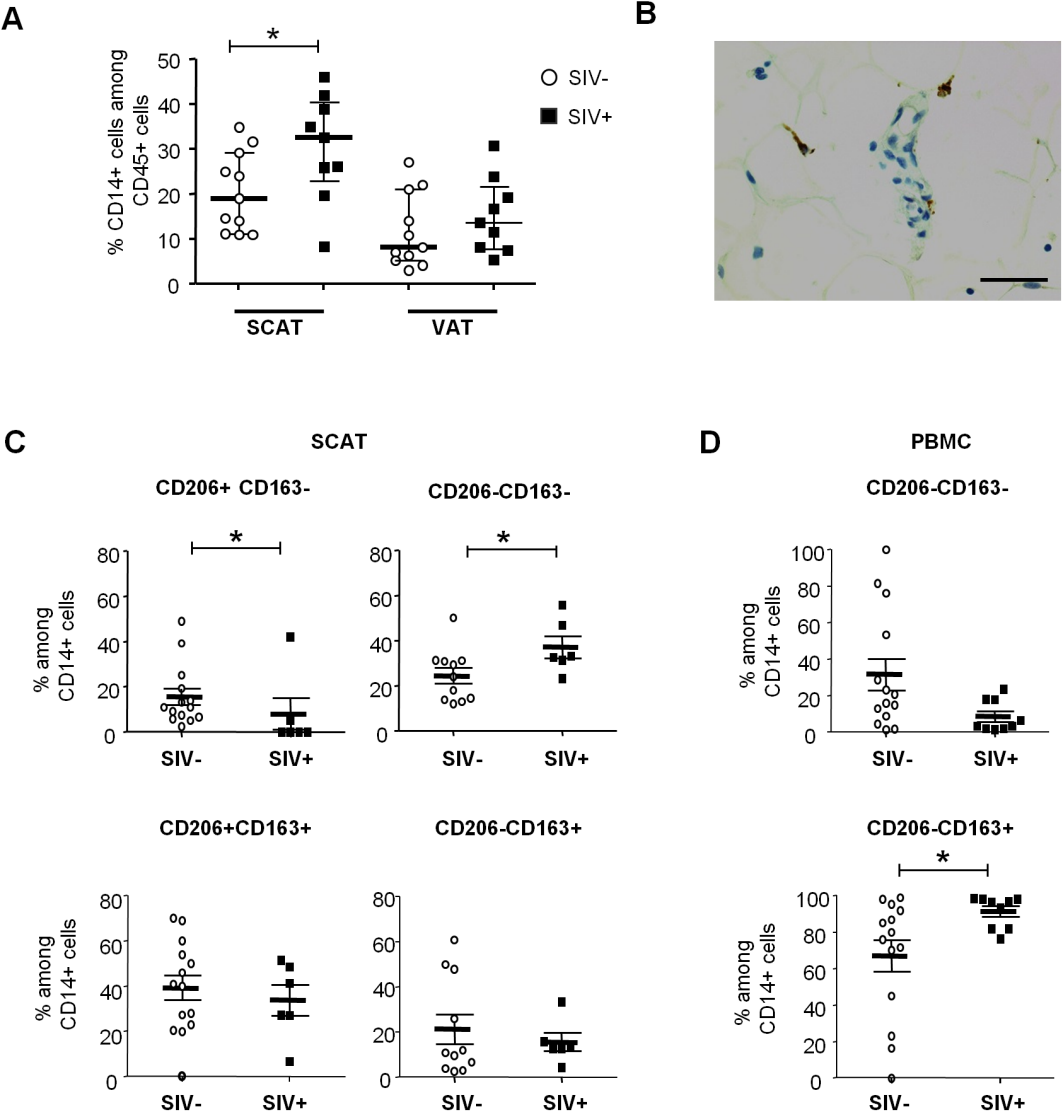
The influence of SIV infection on adipose tissue macrophage numbers and phenotypes. (A) The percentage of CD14-expressing cells among CD45^+^ cells recovered in the SVF from SCAT and VAT from non-infected animals (open circles, n = 13) and SIV-infected animals (filled squares, n = 9). (B) Representative adipose tissue sections, confirming the presence of macrophages in adipose tissue in immunochemical preparations (CD68 staining). Scale bar: 50 μm, magnification x400. (C, D) Analyses of CD206 and CD163 expression on adipose-resident CD14-expressing cells recovered from SCAT (C) and PBMCs (D) from non-infected animals (open circles, n = 11) and SIV-infected animals (filled squares, n = 9). CD206-expressing fractions were not detected in PBMCs. Gating strategies are shown in S4 Fig. Data are quoted as the median [interquartile range]. Significant differences in a Mann-Whitney non-parametric test are shown as * p<0.05.

We next evaluated the changes among adipose macrophages (Fig 5), which are involved both in innate immunity and adipose homeostasis. At present, there is no clear phenotypic strategy for identifying tissue-resident macrophages and defining their activation profile. The pro-inflammatory (M1) versus anti-inflammatory (M2) distinction (commonly used in murine models) may not reflect the great heterogeneity of macrophage phenotypes in tissues. Indeed, macrophages probably develop across a continuum, with anti-inflammatory to pro-inflammatory profiles. In the present study, we considered macrophages to be CD45^+^CD3^-^CD14^+^ cells. The proportion of macrophages among CD45^+^ cells in SCAT was greater in SIV-infected animals than in non-infected animals (Fig 5A). A similar trend was observed in VAT. These findings are in line with the macrophage accumulation previously described in the context of adipose inflammation [26]. We confirmed the presence of macrophages in adipose tissue by performing immunohistochemical analyses (CD68 staining) of tissue sections (Fig 5B).

We next evaluated the activation profile of adipose tissue macrophages by screening for so-called M2 markers (CD206 and CD163) associated with anti-inflammatory activity. Due to the tissue macrophages’ high level of auto-fluorescence, analyses were performed using isotype controls for both CD206 and CD163 staining, and “fluorescence minus one” (FMO) strategies were applied to accurately identify the different subsets (S4A Fig). In non-infected animals, most adipose tissue macrophages were found to express both CD206 and CD163 in SCAT and VAT—suggesting that anti-inflammatory M2 macrophages were predominant in non-infected adipose tissue, as previously reported for humans and mice [24,59] (Fig 5C). Importantly, CD206 expression on CD14-expressing cells was restricted to adipose-resident cells and was not detected in PBMCs. In contrast, the CD206^-^CD163^+^ fraction was predominant in PBMCs (73.9% [62.3%-92.6%]) but extremely rare in SCAT and VAT SVF (S4B Fig).

When considering SIV-infected animals, the CD206^+^CD163^+^ populations were highly predominant in adipose tissue. However, the frequency of the anti-inflammatory CD206^+^CD163^-^ fraction was significantly lower in SIV-infected animals than in non-infected animals for both SCAT (p = 0.0258) and VAT (p = 0.0187) (Fig 5C and S5 Fig). Conversely, the CD206^-^CD163^-^ fraction of adipose-resident macrophages (which presumably corresponds to pro-inflammatory macrophages) was elevated in SIV-infected animals (p = 0.0269). It is noteworthy that opposite changes were observed for CD14-expressing cells in PBMCs (Fig 5D): SIV infection was associated with a lower CD206^-^CD163^-^ fraction and a higher CD206^-^CD163^+^ fraction. Taken as a whole, our results demonstrated that chronic SIV infection skews both adipose tissue T cell and macrophage populations towards a more activated profile.

### Adipose tissue as a site of SIV infection

**Fig 6:**
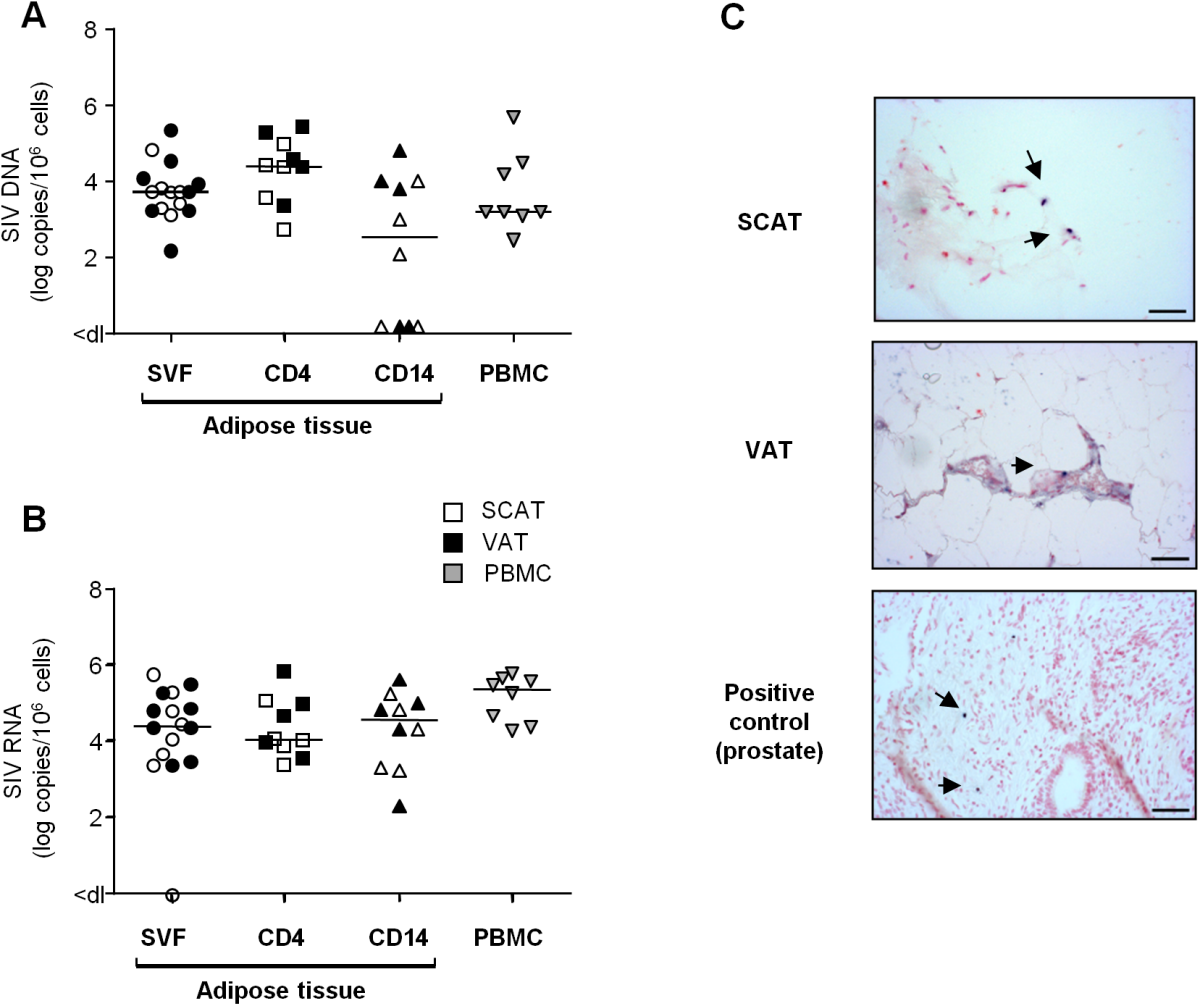
Quantification of SIV DNA and RNA in adipose tissue cells. (A, B) Quantification of SIV DNA (A) and RNA (B) was performed on SVF (n = 8), sorted adipose CD4^+^ T cells (CD4) or CD14-expressing cells (CD14) (n = 5) and PBMCs (n = 8) from SIV-infected animals. SIV DNA and RNA assays were performed in duplicate and the results are expressed in log SIV DNA copies per million cells. Data are quoted as the median [interquartile range]. Significant differences in a Mann-Whitney non-parametric test are shown as * p<0.05. (C) *In situ* hybridization for SIV RNA was performed on SCAT and VAT recovered from one macaque. Prostate tissue from a viremic SIV-infected macaque was used as positive control. One slide was analyzed for each sample. SIV RNA staining is shown at each site. Scale bar: 50 μm.

To date, assays for HIV DNA in adipose tissue have been performed on both whole tissue samples and adipocytes. No consensus has emerged from these studies, although it is currently assumed that adipocytes are not a major target for HIV *in vivo*. In the present work, we focused on the SVF and potential HIV/SIV target cells in particular (CD4^+^ T cells and macrophages). We looked for cell-associated SIV DNA and RNA in total SVF (n = 8) and sorted CD4^+^ T cell and macrophage fractions (n = 5 each) from SIV-infected animals. As shown in Fig 6A, SIV DNA was detected in SVF samples from SCAT and VAT in all animals tested and no significant difference between the two sites was detected. Sorted CD4^+^ T cell fractions from all SCAT and VAT samples (n = 10) were positive for SIV DNA. The observations for adipose CD14^+^ cells were more heterogeneous: 3 of the 5 animals were positive for SIV DNA in both SCAT and VAT. The median SIV DNA levels were 3.7 log DNA copies/10^6^ cells in total SVF and 3.2 log SIV DNA copies/10^6^ cells in PBMCs. Additionally, 4.3 and 2.3 log viral DNA copies/10^6^ cells were detected respectively in the sorted CD4^+^ fraction and CD14^+^ cells recovered from adipose tissue.

We analyzed viral RNA in a total of 11 animals by performing an RT-PCR assay on cell suspensions (n = 8) (Fig 6B) and *in situ* SIV RNA hybridization (n = 3) (Fig 6C). SIV RNA was detected in the SVF from all animals tested (except for one SCAT sample). SIV RNA levels in SVF collected from SCAT and VAT (4.5 log SIV RNA copies/10^6^ cells) were similar to those observed in PBMCs (5.3 log SIV RNA copies/10^6^ cells). Quantitative assays of SIV RNA were also performed in CD4^+^ and CD14^+^ cell subsets from 5 animals. The mean level of SIV RNA (in log copies per million cells) was 4.1 in a sorted adipose CD4^+^ fraction and 4.5 in adipose CD14^+^ cells. *In situ* hybridization was performed on SCAT and VAT samples and confirmed the presence of SIV RNA in both tissues as shown in one of the three animals tested (Fig 6C). We next compared SIV DNA and RNA contents in CD4^+^ T cells and CD14^+^ cells recovered from various tissues/cell sources: adipose tissue, PBMCs and mesenteric lymph nodes (LNs) (S6 Fig). Levels of SIV DNA and RNA within the CD4^+^ T cell fractions were similar in all tissues tested—suggesting that the proportion of CD4^+^ T cells in infected adipose tissue was equivalent to that in other tissues where viral replication is known to occur extensively. The same observation applied to CD14^+^ cells. In conclusion, we demonstrated that the SVF fraction from chronically SIV-infected animals included SIV-infected CD4^+^ T lymphocytes and macrophages. SIV RNA was detected in both the total SVF fraction and adipose CD4^+^ and CD14^+^ fraction—suggesting that viral production occurs in adipose immune cells.

### HIV-infected cells are present in adipose tissue samples from ART-treated, aviremic patients

**Fig 7:**
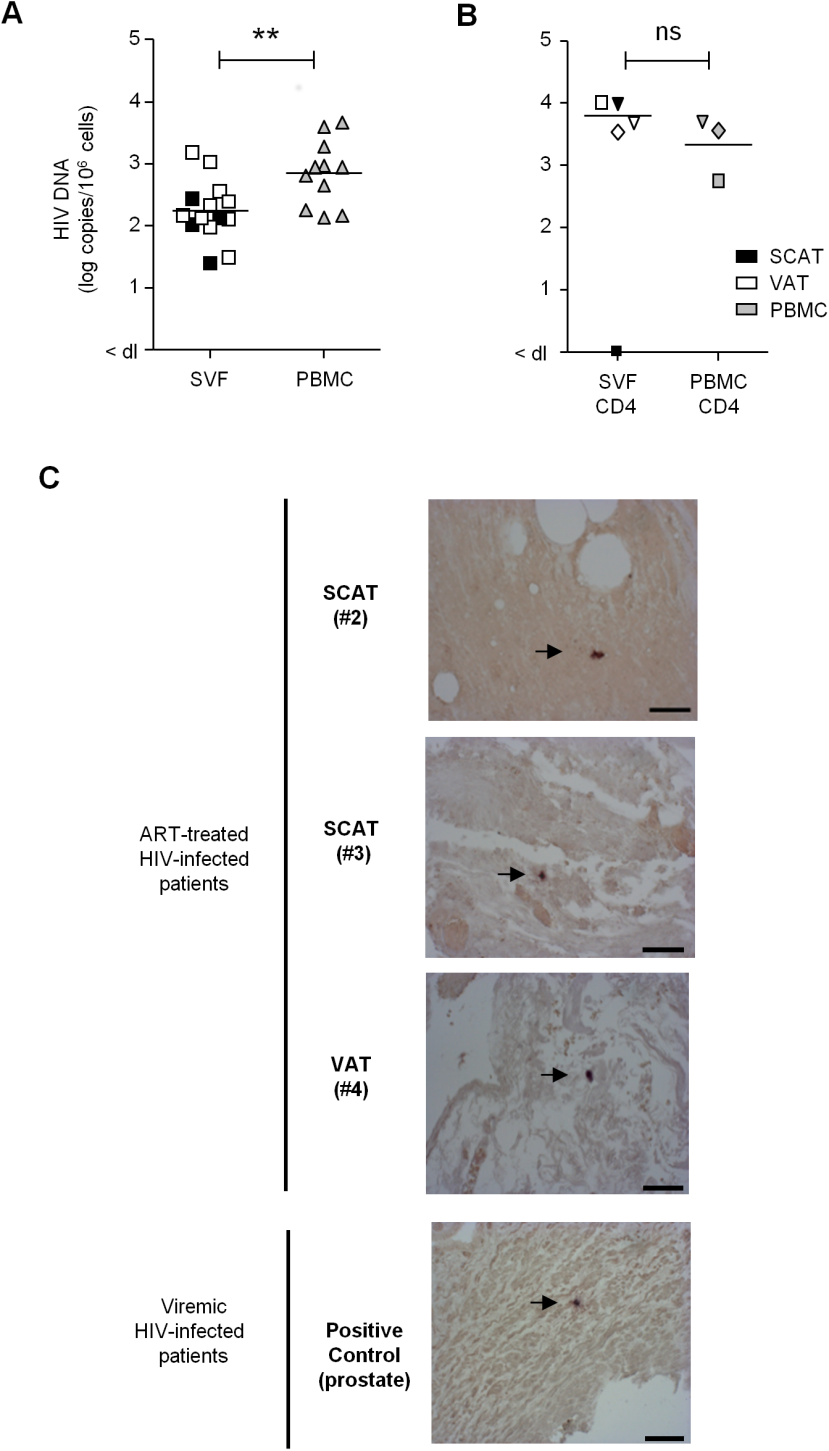
Detection of HIV DNA and RNA in adipose tissue. (A) Detection of HIV DNA in SVF cells and PBMCs from 11 ART-treated, HIV-infected patients. Each patient is represented by a symbol, and the shading represents the type of adipose tissue: SCAT (open symbols), VAT (filled symbols). (B) HIV DNA detection in sorted CD4^+^ T cells recovered from adipose tissue and PBMCs from 3 ART-treated, HIV-infected patients. The HIV DNA detection assay was performed in duplicate and is expressed in log copies per million cells. The detection limit differed as a function of the numbers of cells tested and is indicated as <dl on the graph. (C) An *in situ* hybridization assay for HIV RNA was performed on three samples of adipose tissue recovered from ART-treated, HIV-infected patients (1 VAT and 2 SCAT samples). Positive control: a prostate tissue sample from a viremic patient. One slide was analyzed for each sample. HIV RNA staining for each positive patient is shown. Scale bar: 50 μm.

To ascertain whether adipose tissue may constitute a viral reservoir, we collected adipose tissue samples from 13 ART-treated HIV-infected patients who had undergone elective visceral surgery for non-HIV related causes (Table 1). In 11 patients, we searched for viral DNA in SVF cells and PBMCs. Even after clinically effective ART treatment, HIV DNA was detected in the SVF of all samples tested (11 from VAT and 4 from SCAT) (Fig 7A). The median level of HIV DNA in SVF from VAT (2.15 [2.01–2.47] log DNA copies/million cells) was significantly lower than in PBMCs (2.94 [2.25–3.28]). However, this low level of infection in adipose tissue might primarily reflect the low proportion of hematopoietic cells in adipose tissue. We therefore quantified HIV DNA in sorted CD4^+^ T cells and sorted CD206^+^ CD14^+^ cells from three patients. Contamination by circulating monocytes was controlled for by measuring CD206 expression on CD14^+^ cells. In one sample, HIV DNA could not be detected in adipose CD4^+^ T cells—presumably because of low DNA input. In the samples with detectable virus, the median level of HIV DNA in CD4^+^ T cell fractions recovered from adipose tissue was 3.83 log DNA copies/million cells (compared with 3.56 in sorted peripheral blood CD4^+^ T cells). We failed to detect HIV DNA in the three sorted CD14^+^ CD206^+^ samples tested. Therefore, in patients on long-term ART with no detectable viremia, we confirmed that HIV-infected cells are present in the SVF (mainly tissue-resident CD4^+^ T cells). Given that the detected HIV DNA might have been replication-defective, we also screened for viral RNA in adipose tissue sections using *in situ* hybridization. Two SCAT samples and one VAT sample were obtained from ART-treated HIV-infected patients. As shown in Fig 7C, cells expressing HIV RNA were detected (albeit in low numbers) in the three adipose tissue samples studied. We therefore demonstrated that productive, HIV-infected cells are present in the adipose tissue of ART-treated patients.

**Table 1:** Study population.

ID	Gender	Age (year)	CD4 Nadir (/μL)	Treatment duration (year)	Treatment at sampling	VL at sampling (RNA copies/mL)	CD4 count at sampling (/μL)
**1**	M	41	55	5	tenofovir/ emtricitacine/ darunavir	<40	272
**2**	M	45	371	4	Efavirenz/ tenofovir / emtricitabine	<40	371
**3**	F	48	23	22	Raltegravir/ abacavir/ lamuvidine/ darunavir	<40	381
**4**	F	34	262	11	Truvada/ darunavir	<20	525
**5**	F	47	280	16	Zidovudine/ lamivudine/ nevirapine	<20	788
**6**	F	34	162	6	Zidovudine/ lamivudine/ nevirapine	<40	162
**7**	M	48	206	8	Rilpivirine/Tenofovir/Emtricitabine	<20	464
**8**	M	48	296	5	Efavirenz/ tenofovir / emtricitabine	<40	673
**9**	M	75	177	18	Truvada/nevirapine	<20	423
**10**	F	75	NA	20	Tritherapy not communicated	<40	NA
**11**	F	38	3	16	zidovudine/lamivudine/ lopinavir	<40	597
**12**	F	51	80	26	Truvada/ fosamprenavir/ ritonavir	<40	232
**13**	F	38	237	16	Truvada/ darunavir/ ritonavir	<40	613
**Median and interquartile range**							
	**8F/5M**	**47**	**192**	**16**		**<40**	**444**
		[38–49,5]	[61–276]	[5–19]			[297–609]

**Fig 8:**
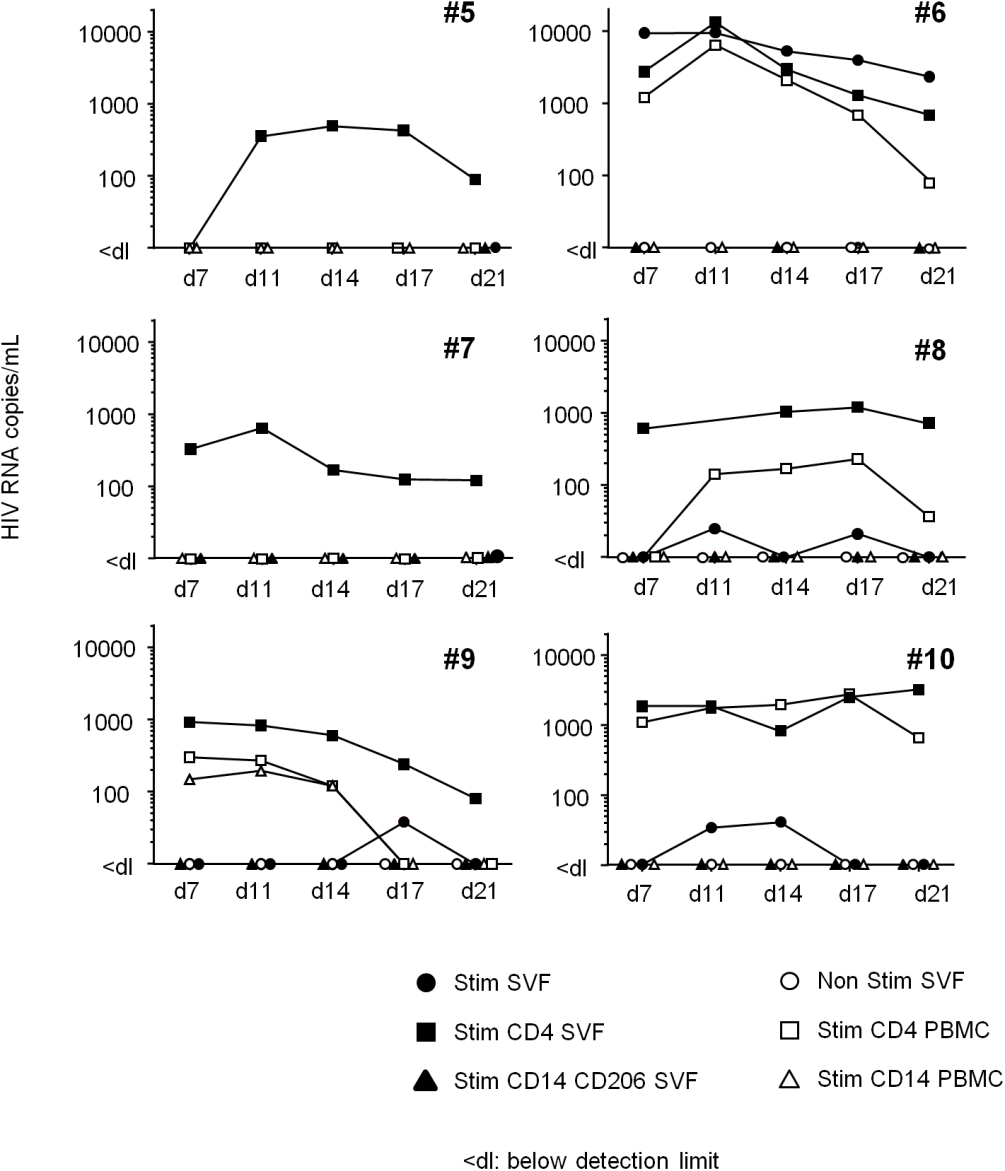
HIV RNA production following *in vitro* reactivation. Total SVF (filled circles), sorted CD4^+^CD3^+^ (filled squares) and CD14^+^CD206^+^ cells (filled triangles) from adipose tissue, and sorted CD4^+^CD3^+^ (open squares) and CD14^+^ cells (open triangles) from PBMCs were co-cultured with allogeneic, pre-activated CD4^+^ T cells and then stimulated. The cell number differed according to the cell fraction: 1x10^6^ for the total SVF fraction, 1x10^5^ to 5x10^5^ for sorted CD4^+^ T cells, and 5x10^4^ to 2x10^5^ for sorted, CD14-expressing cells. HIV RNA was detected in supernatants collected at D7, 11, 14, 17 and 21. As a negative control, 1x10^6^ SVF cells were cultured in the absence of allogeneic, pre-activated CD4^+^ T cells (Ns SVF open circles). Samples from six ART-treated HIV infected patient were tested and the HIV RNA detection assay was performed five times for each sample.

The *ex vivo* induction of viral replication in sorted cells is a further means of demonstrating that HIV is replication-competent. In six long-term ART-treated patients, we performed an *in vitro* viral reactivation assay on total SVF cells, sorted adipose CD4^+^ and CD206^+^CD14^+^ cells and sorted peripheral blood CD4^+^ or CD14^+^ cells in the presence of allogeneic pre-activated CD4^+^ T cells and phytohemagglutinin (Fig 8). In order to obtain sufficient numbers of cells, SCAT and VAT samples were pooled when both were available. Although small numbers of cells (5 10^4^ to 5 10^5^) were used for this reactivation assay, we detected the induction of viral replication in the sorted CD4^+^ T cell fraction from the SVF of all six patients. HIV RNA in supernatants from the *ex vivo*-activated, sorted SVF CD4^+^ T cells was detected from day 7 to day 21 in nearly all patients. In two patients (#5, 7), HIV RNA was detected solely after the reactivation of sorted SVF CD4^+^ T cells but not in total SVF or in PBMCs—probably because of the small number of infected cells in the culture. In the four other patients (#6, 8, 9, 10), HIV RNA was detected in similar numbers of *ex vivo*-activated CD4^+^ T cells recovered from both adipose tissue and PBMCs. Induction of HIV replication from SVF was detected in these four patients (albeit at low levels in three—probably due to the small number of infected cells). No HIV RNA was detected in cultures of activated adipose CD14^+^CD206^+^ fractions. Importantly, HIV RNA was not detected in non-activated SVF cell cultures (except in one patient), meaning that we could rule out the amplification of non-latent, pre-existing HIV. Our results show that adipose CD4^+^ T cells were infected by replication-competent HIV.

In conclusion, we performed three levels of detection: (i) HIV DNA using PCRs, (ii) HIV RNA using *in situ* hybridization and (iii) HIV RNA in supernatants in an *in vitro* reactivation assay based on ultrasensitive RT-PCR. We demonstrated that latently HIV-infected cells were present in the SVF from both SCAT and VAT in 6 ART-treated patients. HIV DNA was consistently detected in adipose CD4^+^ T cells—strongly suggesting that adipose tissue is an HIV reservoir in aviremic patients on long-term ART.

## Discussion

Residual chronic inflammation and viral persistence are two key features of ART-treated, HIV-infected patients. Although several mechanisms have been described, the establishment and persistence of low-grade inflammation are not fully understood and remain to be characterized. Given that adipose tissue is a major site of inflammation in a context of obesity, we hypothesized that the adipose tissue changes associated with HIV infection may drive low-grade inflammation. Indeed, the literature data reveal a strongly pro-inflammatory profile of adipose tissue during HIV infection—although most of these studies were performed on ART-treated patients [36,41,60]. In order to rule out the potential confounding role of ART and to collect sufficient amounts of tissue, we studied the SIV/macaque infection model. Chronic SIV infection *in vivo* was associated with a markedly higher adipocyte density (i.e. cell numbers per field) and SVF cell count (expressed per gram of adipose tissue). The elevated adipocyte density has been linked to an abnormally low adipocyte size, which in turn may reflect drastic alterations of adipogenesis [61,62] and/or lipolysis [48,60]. The massive increase in SVF cells in adipose tissue in chronically infected animals essentially reflected the accumulation of CD45^-^ cells. The CD45^-^ cells recovered in the SVF of infected animals need to be identified more accurately. Those in the SVF included pre-adipocytes, the accumulation of which may be related to changes in adipocyte differentiation. These striking changes in CD45- proportion need to be further investigated and could relate to multiple defects affecting adipose tissue during SIV infection such as lipodystrophy, inflammation or viral persistence. The SVF also contains mesenchymal stem cells [63–65], and it remains to be established whether these cells can also be infected. When focusing on immune cells, there was no major change in total leukocyte counts. However, the percentage of CD4^+^ T cells was severely lower in adipose tissue from SIV-infected animals than in non-infected animals, whereas the CD8^+^ T cell percentage was markedly higher. We demonstrated that the change in the CD4/CD8 ratio reflected the accumulation of CD8^+^ T cells, in accordance with previous reports of massive CD8 accumulation during adipose tissue inflammation [27]. Intense recruitment of CD8^+^ T cells was confirmed by the perivascular location of CD8^+^ T cells. However, a direct impact of SIV in adipose tissue cannot be ruled out [53,54].

The fact that CD4^+^ T cell counts were generally unaffected was not expected in the context of SIV infection—especially given the high percentage of central memory CD4^+^ T cells (a major HIV/SIV target) in adipose tissue. Organ-dependent differences in the direction and amplitude of cell depletion have been described [66,67]: CD4^+^ T cell depletion is rapid and massive in the intestinal mucosa, whereas the follicular helper subset (also a major HIV target) even expands in secondary lymphoid organs [68]. Importantly, CD4^+^ T cells were mainly found recovered in the vicinity of adipocytes and far from capillaries. This remote site may favor viral persistence, as was recently described in follicular CD4^+^ T cells [69]. It thus appears that adipose tissue may constitute an additional site for the accumulation of CD4^+^ T cells. Phenotypic analysis revealed that the Tcm CD4^+^ T cells fraction was predominant in both SCAT and VAT, thus confirming the high potential for viral persistence of adipose tissue. The predominance of central memory CD4^+^ T cells, which usually identifies inductive lymphoid site, also raises the question of the lymphoid definition of the adipose tissue: Does adipose tissue constitute a lymphoid site and if so is it an inductive or effector lymphoid site?

We also found that phenotypic changes can be detected in both lymphocytes and macrophages during chronic SIV infection of adipose tissue. Expression levels of the activation marker HLA-DR on adipose tissue T lymphocytes were higher in SIV-infected animals. Macrophages displayed a moderate change in phenotype. SIV infection was associated with an increase in the proportion of CD206^-^CD163^-^ adipose tissue macrophages and a concomitant decrease in the CD206^+^CD163^-^ fraction—suggesting a shift from an anti-inflammatory profile towards a more activated profile or the recruitment of migrating blood monocytes. Overall, we observed features commonly associated with obesity-related inflammation: elevated SVF numbers, the specific recruitment of CD8^+^ T cells, a higher proportion of macrophages and greater expression of activation markers. These observations strongly suggest that adipose tissue is involved in inflammation during SIV infection.

We next investigated the mechanisms that might underlie the increase in adipose tissue inflammation in chronically SIV-infected animals. There is increasing evidence to show that HIV infection *per se* interferes with adipose tissue homeostasis, although the mechanisms remains to be defined [34]. Adipose inflammation may be a consequence of systemic inflammation, with in turn is worsened by adipose inflammation in a vicious circle. Adipose inflammation may also be related to the spread of viral proteins [48–50], CD4^+^ T cell lymphopenia [70] and/or microbial translocation [71,72], all of which are known to alter adipocyte homeostasis. Furthermore, SIV may directly infect adipose-resident immune cells. Importantly, previous reports essentially failed to demonstrate consistent infection of adipocytes and did not provide any information on adipose immune cells. In the present study, SVF fractions were positive for SIV DNA and SIV RNA in all animals tested. SIV DNA was also detected in all sorted adipose CD4^+^ T lymphocytes. SIV DNA was detected less consistently in sorted CD14-expressing cells (3 out of 5 in both SCAT and VAT). The results for cell-associated SIV RNA essentially corroborated those for SIV DNA. We demonstrated that in SVF fractions, SIV was consistently present in CD4^+^ T lymphocytes and less frequently present in macrophages. Our detection of viral DNA and RNA in stromal vascular cells collected from adipose tissue of chronically viremic animals thus confirms that adipose tissue is a site of infection.

We next sought to confirm the presence of viral infection in human adipose tissue and, more importantly, to characterize viral persistence in ART-suppressed HIV-infected patients. Three different assays were performed: (i) detection of viral DNA in SVF (in 11 patients) and in sorted CD4^+^ T cell fractions (from 3 patients), (ii) *in situ* RNA hybridization on fixed sections of adipose tissue (in 3 patients), and (iii) *in vitro* viral reactivation (in 6 patients). HIV DNA was detected in all SVF samples tested; this finding is in line with Couturier et al.’s report of HIV DNA in the SVF of 5 ART-treated, HIV-infected patients [73]. Importantly, we were able to detect HIV DNA in sorted adipose CD4^+^ T cell fractions but not in CD206^+^ CD14-expressing cells. It is noteworthy that sorted CD4^+^ T cells recovered from adipose tissue had much the same levels of HIV DNA as PBMCs. These results suggest that the proportion of infected cells is similar in adipose tissue CD4^+^ T cells and in peripheral blood CD4^+^ T cells. In contrast to the situation in chronically viremic macaques (in which SIV DNA was detected in a CD14^+^ fraction recovered from the SVF), we did not detect HIV DNA in CD14^+^CD206^+^ cells recovered from the SVF in aviremic patients. We checked whether this discrepancy was related to the difference in macrophage selection (i.e. CD14^+^ cells vs. CD14^+^CD206^+^ cells). CD14^+^CD206^+^ cells sorted from viremic macaques were still positive for SIV DNA, suggesting that viral DNA may be present in macrophage subsets in viremic stages but might not persist during long-term ART. One can reasonably presume that the half-life of macrophages is shorter than that of Tcm CD4^+^ T cells. Alternatively, the small number of macrophages collected may have prevented us from detecting a small proportion of infected cells. This aspect will be investigated further. Lastly, six samples were reserved for a viral replication assay. Although only small numbers of sorted adipose cells were available, we were able to monitor *in vitro* viral replication by detecting HIV RNA in supernatants after the incubation of adipose tissue CD4^+^ T cells with allogeneic pre-activated CD4^+^ T cells. HIV RNA was consistently detected in cultures of CD4^+^ T cells sorted from the SVF, suggesting that adipose CD4^+^ T cells are infected by replication-competent HIV. In four patients, *ex vivo* HIV replication was induced to the same extent in sorted CD4^+^ T cells from the SVF and from PBMCs. These results suggest that the proportion of infected cells is much the same among adipose tissue CD4^+^ T cells and peripheral blood CD4^+^ T cells; this hypothesis is supported by the fact that sorted CD4^+^ T cells from adipose tissue and PBMCs had similar HIV DNA contents. In contrast, replication-competent HIV was detected in the SVF only in two patients. The high frequency of memory CD4^+^ T cells in adipose tissue (relative to PBMCs) may explain the high proportion of latently infected cells. Otherwise the number of latently infected CD4^+^ T cells in adipose tissue might be higher than in PBMCs in some patients on ART. These findings emphasize the need to sample several tissues when studying HIV reservoirs in patients on ART. Further investigation (using limiting dilution assays) is necessary but would be technically challenging (given the low numbers of resident tissue CD4^+^ T cells in patients).

Overall, we were able to demonstrate the persistence of HIV DNA and RNA within stromal vascular cells in adipose tissue recovered from ART-treated HIV-infected patients. These observations were supported by the results of *in vitro* reactivation experiments, showing that adipose CD4^+^ T cells contained replication-competent HIV. This dataset strongly supports the hypothesis whereby adipose tissue constitutes an important viral reservoir. Adipose tissue may thus constitute a favorable environment for viral persistence for several reasons: (a) constant inflammation favors viral replication, (b) the presence of elevated fractions of activated and central memory CD4^+^ T cells, which are HIV’s natural targets, (c) the potentially insufficient distribution of some antiretroviral drugs into adipose tissue [44], which may favor viral persistence, and (d) the specific metabolic and immune activity of adipose tissue, which may affect the effectiveness of immune responses [74].

In the present work, our analyses of SCAT and VAT from SIV-infected macaques and ART-suppressed patients, showed that (i) SIV infection induced immune activation and a pro-inflammatory profile in adipose tissue immune cells and (ii) these immune cells were indeed infected by SIV/HIV. These results indicate that adipose tissue constitutes a new, relatively large reservoir for the virus that could be involved in chronic immune activation and low-grade inflammation. Our observations have major implications in the context of HIV disease. Firstly, they emphasize the crucial requirement for the broad diffusion of antiretroviral drugs within tissues; combination therapy must include drugs that diffuse not only into adipose tissues but also into tissue CD4^+^ T cells and macrophages. Two main mechanisms may prevent efficient activity of ART on adipose infected cells: (i) low accessibility of ART to fat tissue [44], (ii) sequestration of drugs inside the lipid droplets at the expense of adipose infected immune cells [75]. Secondly, they provide an interesting rationale for the use of drugs with metabolic activity. It might be interesting to reconsider the anti-inflammatory impact of statins [76,77] by focusing on adipose sites and addressing the drugs’ potential impact on viral reservoirs. Thirdly, gender differences in adipose tissue distribution, the inflammatory profile and immune cell content [78] may underlie differential susceptibility to the establishment of viral reservoirs. Fourthly, our results open up new therapeutic strategies for limiting the size of viral reservoirs, chronic inflammation and associated comorbidities (via the modulation of adipose tissue related pathways rather than strictly immune pathways).

## Materials and Methods

### Animals, infection and sample collection

Twenty-three adult cynomolgus macaques (*Macaca fascicularis*) were infected via the intravenous, intravaginal or intrarectal route with 0.5 to 5,000 50% animal infectious doses (AID50) of SIVmac251 biological isolate and monitored for 15 months (median value, interquartile range: [11–18]). SIV infection in cynomolgus macaques closely recapitulates the major features of HIV infection, including progression towards AIDS. At sacrifice, the plasma viral load in SIV-infected animals was 4.4x10^4^ [0.7x10^4^-6.7x10^4^] RNA copies/mL. Twenty one non-SIV-infected animals were used as controls. At sacrifice, blood samples and 10 to 35g samples of abdominal subcutaneous adipose tissue (SCAT) and visceral adipose tissue (VAT) were collected. Adipose tissues were devascularized to prevent blood contamination. PBMCs were isolated from EDTA-anticoagulated blood by Ficoll density gradient centrifugation.

### Ethics statement

Adult cynomolgus macaques were imported from Mauritius and housed in the animal facility at the *Commissariat à l’Energie Atomique et aux Energies Alternatives* (CEA, Fontenay-aux-Roses, France). Non-human primates (NHPs, which include cynomolgus macaques) are housed and handled in accordance with French national regulations and subject to inspection by the veterinary authorities (CEA Permit Number A 92-032-02). The CEA facility complies with the Standards for Human Care and Use of Laboratory of the Office for Laboratory Animal Welfare (OLAW, USA) under OLAW assurance number #A5826-01. The use of NHPs at CEA is also in line with the European Directive (2010/63, recommendation Nu9). The animals were used under the supervision of the veterinarians in charge of the animal facility. The study protocols were reviewed by the CEA’s Animal Care and Handling Committee (*Comité d’Ethique en Expérimentation Animale*, registered with the French Ministry of Research). Animals were housed in adjoining, individual cages (allowing social interactions) and under controlled humidity, temperature and light conditions (12-hour light/12-hour dark cycles). Water was available ad libitum. Animals were monitored and fed with commercial monkey chow and fruits once or twice daily by trained personnel. Macaques were provided with environmental enrichment, including toys, novel foodstuffs and music under the supervision of the CEA’s Animal Care and Handling Committee.

After sedation with ketamine chlorhydrate, animals were sacrificed by intracardiac injection of sodium pentobarbital (Vetoquinol, Paris, France; 180 mg/kg).

### Human samples

All 13 patients provided their written, informed consent to participation. The study protocol was approved by the regional investigational review board (*Comité de Protection des Personnes Ile-de-France VII* (Paris, France)). Adipose tissue samples from 13 ART-treated HIV-1-infected patients were recovered during elective abdominal surgery for non-AIDS-related indications. SVF was isolated from fresh samples. The 13 patients were on long-term ART and had displayed an undetectable viral load for over four years. In eleven patients, SVF samples were screened for HIV DNA. Five SVF samples were reserved for an HIV DNA assay in sorted fractions and 6 were reserved for the *in vitro* reactivation assay. *In situ* hybridization was performed on fixed adipose sections from three patients on ART and on a prostate sample (from a viremic patient) as a positive control.

### Isolation of the stromal vascular fraction (SVF)

SCAT and VAT were weighed, washed twice in PBS 1X 5% fetal bovine serum (FBS), cut into pieces of 2 to 3 mm and then digested in a bath of collagenase (C2139, Sigma) at a concentration of 0.33 mg /mL in DMEM supplemented with 5% FBS for 30 min at 37°C with constant shaking. Mechanical dissociation by suction/discharge with a 10 mL syringe was then performed. Next, the adipose suspension was filtered through a 100 micron mesh. Following an initial low-speed centrifugation (300*g*, 10 min), adipocytes (the upper phase) were separated and the lower phase (comprising the SVF cells) was centrifuged further. After two additional washes, the pellet containing the SVF was resuspended in PBS with 5% FBS. SVF cell suspensions were then counted in Malassez counting chambers (C-chip, NanoEntek, Seoul, Korea) under the microscope, using Trypan blue to exclude dead cells. SVF was either directly frozen for molecular analyses, frozen in FBS 10% DMSO for cell preservation, and/or sorted or analyzed immediately by flow cytometry.

### Phenotypic characterization of macrophages and T lymphocytes from adipose tissue

Staining was performed after the saturation of Fc receptors by incubation with FC block (BD), mouse serum (eBiosciences) and healthy macaque serum (an in-house preparation) for 30 min at 4°C. Amine-reactive blue dye (Live/dead Fixable, Life Technologies) was used to assess cell viability and exclude dead cells from the analysis. Cells were stained with monoclonal antibodies (incubation for 15 min at 4°C), washed in PBS 1X/10% FBS and fixed in commercial fixative solution (CellFIX, BD Biosciences).

A variety of antibody panels were used to study adipose tissue T lymphocytes and macrophages [55]. Macrophages were identified using the following panel: CD16 V450 (Clone 3G8)/ CD206 FITC (Clone 19.2)/ CD14 PerCPCy5.5 (M5E2)/ CD163 APC (GHI/61)/ CD11b PECY7 (BEAR1)/ HLA-DR PECF574 (G46-6)/ CD3 AF700 (SP34-2). Corresponding isotype controls for CD163 and CD206 were used at the same concentrations as the reference antibody, in accordance with the manufacturer’s instructions. Lymphocyte subsets were analyzed as follows: CD45 V500 (D058-1283 for macaques, HI-30 for humans)/ CD4 PerCPCy5.5 (L200) / CD8 V450 (leu2a)/ CD95 APC (DX-2)/ CD28 PE (CD28.2) / HLA-DR PECY7 (G46-6)/ CD20 AF700 (2H7)/ Ki-67 FITC (B56). Additional combinations with CCR5 APC (3A9), CD69 PECY7 (FN50) antibodies were also applied. All antibodies were purchased from BD Biosciences, with the exception of anti-CD11b (Beckman-Coulter). Data were acquired with an LSR Fortessa cell analyzer (BD Biosciences) and analyzed with FlowJo software (Treestar).

### Microscopy and immunochemistry

Samples of SCAT and VAT were fixed in 4% buffered formalin and embedded in paraffin. Sections (thickness: 3 microns) were stained with hematoxylin/eosin/saffron reagent. Adipocytes were counted in an average of 10 high-power fields (HPFs). Parallel sections were immunostained for CD3 (clone F.2.38, Dako), CD4 (clone 4B12, Leica Biosystem), TIA1 (TIA1 cytotoxic granule-associated RNA binding protein, clone 2G9, Immunotech) and CD68 (clone PG-M1, Dako), in order to identify CD4^+^ T lymphocytes, cytotoxic T cells and macrophages. Unless otherwise stated, intravascular leucocytes were excluded from counting, although T cells located in the vicinity of capillaries or far from capillaries were also studied.

### Sorting of adipose CD4^+^ T cells and CD14^+^ macrophages

SVF cells from SCAT and VAT were stained as described above, using amine-reactive blue dye to identify the dead cells. CD4^+^CD3^+^CD45^+^L/D^-^ cells (CD4^+^ T cells) in macaques and humans, CD14^+^CD3^-^CD20^-^CD45^+^L/D^-^ cells in macaques and CD14^+^CD206^+^CD3^-^CD20^-^CD45^+^L/D^-^ cells (macrophages) in humans were sorted on a FACS ARIA cell sorter (BD Biosciences). The gating strategy for macrophage isolation used CD14 fluorescence minus one (FMO) control staining to eliminate contamination due to potential auto-fluorescence of SVF cells. Sorting purity profile are presented in S7 Fig. In both NHPs and humans, sorting purity was consistently greater than 95% (CD14-expressing cells: 97.2% [95.1–98.7], CD4^+^ T cells: 96.9% [96.1–99.1]).

### SIV DNA and RNA detection

RNA and DNA were extracted from cells as follows: 1x10^6^ PBMCs or SVF cells and various numbers of sorted CD4^+^ and CD14^+^ cell numbers (ranging from 50000 to 200000) were resuspended in 350 μL of RLT buffer (Qiagen, France). 200 μL of TE buffer were added. Next, NaCl (5M) was added. The mixture was incubated at 4°C for between 15 and 60 min, followed by centrifugation for 15 min at 3000 rpm and 4°C. RNA was precipitated from the supernatant using pH 4.5 phenol/chloroform (Sigma-Aldrich) at a v/v ratio of 5:1. 200 μl of TE buffer was added to the pellet. DNA was then precipitated from the mixture in phenol/chloroform/isoamyl alcohol 25:24:1 (pH = 8, Sigma Aldrich). Cell-associated viral DNA and RNA were measured by qPCR as described previously, using primers and probes designed specifically for SIVmac251 isolates [79]. PCRs were performed in duplicate; the detection limits were 35 SIV DNA copies/10^6^ cells and 50 SIV RNA copies/10^6^ CCR5 copies. Only reactions for which duplicates gave consistent results were included. Positive and negative controls were used to rule out sample contamination. SIVmac plasmids were used as standards to calculate SIV DNA copy numbers. Primers and plasmids for CCR5 were used to normalize the viral levels against the number of cells [80]. Plasma SIV RNA was quantified as previously described [81].

### Ultrasensitive total HIV-DNA and HIV-RNA quantification

Total HIV DNA was quantified in an ultrasensitive real-time PCR assay of frozen SVF cells, sorted adipose CD4^+^ and CD14^+^ cells in SVF, and PBMCs using the GENERIC HIV-DNA assay from Biocentric (Bandol, France), as previously described [82,83]. Total DNA was extracted with a QIAamp All prep DNA/RNA microkit or minikit (Qiagen), depending on the number of cells available (less than and more than 1 million cells, respectively). The entire HIV-DNA extract was tested in two to four replicates, with a threshold of 2 HIV-DNA copies per PCR. The thresholds varied according to the available cell numbers and were calculated for each sample. 1x10^6^ cells were analyzed for total SVF and PBMC samples. Sorted SVF CD4^+^ and CD14^+^CD206^+^ cell numbers were consistently lower (from 14000 to 320000), and so the detection limit was set to <3.23 log copies/10^6^ cells. Following *in vitro* reactivation, HIV-RNA was quantified in culture supernatants using an ultrasensitive real-time PCR assay (GENERIC HIV, Biocentric, Bandol, France). The extracts were tested in two to five replicates [84].

### *In situ* hybridization

Paraformaldehyde-fixed, paraffin-embedded tissues were assayed for SIVmac and HIV-1 RNA expression using a digoxigenin-antidigoxigenin technique, as previously described [67,85]. The digoxigenin-UTP-labeled riboprobe spanned the whole SIVmac or HIV-1 genome (Lofstrand Labs Ltd, Gaithersburg, MD, USA). Nitroblue tetrazolium-5-bromo-4-chloro-3-indollylphosphate toluidinium revelation was used to detect infected cells in the tissues. The specificity of the hybridization signal was always checked by hybridizing sense probes on parallel sections and anti-sense probes on non-infected adipose tissues. Prostate tissues from a viremic SIV-infected macaque and from HIV-infected patients were used as positive controls, as previously described [86–88].

### *In vitro* reactivation assay

Cell fractions recovered from ART-treated, HIV-infected patients were stimulated with phytohemagglutinin (0.3 μg/mL, Sigma), IL-2 (25 μg/mL, Immunotools) (for SVF and T cell fractions) and LPS (1 μg/mL, Sigma) (for CD14-expressing cells) and co-cultured with allogeneic pre-activated CD4^+^ T cells at a ratio of 1:2.5. Allogeneic CD4^+^ T cells were purified from PBMCs from healthy donors by positive selection using magnetic beads (Miltenyi). At day (D)7 and D14, reactivation was sustained by novel addition of pre-activated CD4^+^ T cells. Supernatants were collected at D3, 7, 11, 14, 17 and 21 and cell pellets were collected at D21. The number of cells used in the reactivation assay depending on the cell fraction isolated from adipose tissue SVF: 1x10^6^ for the total SVF fraction, 1x10^5^ to 5x10^5^ for sorted CD4^+^ T cells, and 5x10^4^ to 2x10^5^ for sorted, CD14-expressing cells. As negative controls, 1x10^6^ SVF cells were cultured in the absence of allogeneic, pre-activated CD4^+^ T cells.

### Statistical analysis

Data are quoted as the median [interquartile range]. Statistical analyses were carried out with GraphPad Prism 5.03 software (GraphPad Software Inc.). A Mann-Whitney non-parametric test was used to compare data from SIV-infected and non-infected macaques. A Wilcoxon matched-pair signed rank test was used to compare different tissues from the same animal. In graphs, the thresholds for statistical significance are indicated as follows: * p<0.05; ** p<0.01, *** p<0.001.

## Supporting Information

S1 Fig The influence of SIV infection on CD8^+^ T cell differentiation. Representative dot plots showing the gating strategies used to define Tn, Tcm, Ttm and Tem subsets among CD8 T cells (based on CD28, CD95 and CCR5 staining) in SCAT and VAT. The right-hand panels show the distribution of CD8^+^ T cells among the different subsets in non-infected animals (n = 5, open column) and SIV-infected animals (n = 7, filled column). Data are quoted as the median [interquartile range]. A Mann-Whitney non-parametric test was used.
(TIF)

S2 Fig Differences in cell composition and phenotype between SVF cells and PBMCs. (A) The percentages of Tn CD4^+^ T cells (CD95^+^CD28^int^) in SCAT, VAT and PBMCs from pooled infected animals (n = 8) animals and non-infected animals (n = 8). (B) The percentage of Tm CD4^+^ T cells (CD95^+^CD28^+^CCR5^+/-^) in SCAT, VAT and PBMCs from infected and non-infected animals. (C) The percentage of CD20-expressing cells among the CD45^+^ fraction in SCAT, VAT and PBMCs from pooled infected animals (n = 5) and non-infected animals (n = 7). Datasets from SIV-infected animals (n = 7) and non-infected animals (n = 5–7) were pooled, since there was no apparent difference between the two groups.
(TIF)

S3 Fig The lack of a significant influence of SIV infection on Ki67 expression on adipose tissue T cells. The percentage of Ki67-expressing cells among CD4^+^ and CD8^+^ T cells recovered from SCAT and/or VAT from 7 infected animals (filled symbols) and 7 non-infected animals (open symbols). Values from peripheral blood T cells are shown when available (n = 5–6).
(TIF)

S4 Fig Difference in the proportion of CD14-expressing cells and their phenotype in SVF cells and PBMCs. (A) Dot plots showing the co-expression of CD206 and CD163 on CD14-expressing cells. Due to high levels of auto-fluorescence, the gating strategy was defined using isotype controls for either anti-CD163 or anti-CD206 antibodies and an unstained approach for the CD163 and CD206 fractions. (B) Graphs showing the percentage of each fraction in CD14-expressing cells recovered from SCAT, VAT and PBMCs from 8 non-infected animals. Similar observations were made in SIV-infected animals. Data are quoted as the median [interquartile range]. Significant differences in a Mann-Whitney non-parametric test are shown as * p<0.05; ** p<0.01; *** p<0.001.
(TIF)

S5 Fig The influence of SIV infection on macrophage phenotype in VAT. Expression of CD206 and CD163 on adipose-resident CD14-expressing cells recovered from VAT from non-infected animals (open circles, n = 8) and SIV-infected animals (filled squares, n = 6). Gating strategies are shown in S4 Fig. Data are quoted as the median [interquartile range]. Significant differences in a Mann-Whitney non-parametric test are shown as * p<0.05.
(TIF)

S6 Fig Comparison of SIV DNA and RNA contents in CD4^+^ T cells and CD14-expressing cells from different tissues (adipose tissue, PBMCs and lymph nodes). Comparison of SIV DNA and RNA levels in sorted CD4^+^ T cell and CD14^+^ cell fractions recovered from different organs (adipose tissue, PBMCs and lymph nodes) in four SIV-infected animals. SIV DNA and RNA assays were performed in duplicate and the results are expressed in log SIV DNA copies per million cells. Due to the low numbers of CD14^+^ cells recovered from lymph node, CD14^+^ cells were sorted from spleen for two animals. Data are quoted as the median [interquartile range]. A Mann-Whitney non-parametric test was used.
(TIF)

S7 Fig Sorting purity. A representative sorting strategy used for CD14^+^ cells and CD4^+^ T cells. Dot plots of cells from a non-infected animal before and after sorting are shown. In both NHPs and humans, sorting purity was consistently over 95% (CD14^+^ cells: 97.2% [95.1–98.7], CD4^+^ T cells: 96.9% [96.1–99.1]).
(TIF)
