# Supplementary figures and images for: Adipose Tissue Is a Neglected Viral Reservoir and an Inflammatory Site during Chronic HIV and SIV Infection
Source: PLoS Pathog. 2015 Sep 24;11(9):e1005153. doi: 10.1371/journal.ppat.1005153 (PMC4581628; doi:10.1371/journal.ppat.1005153)

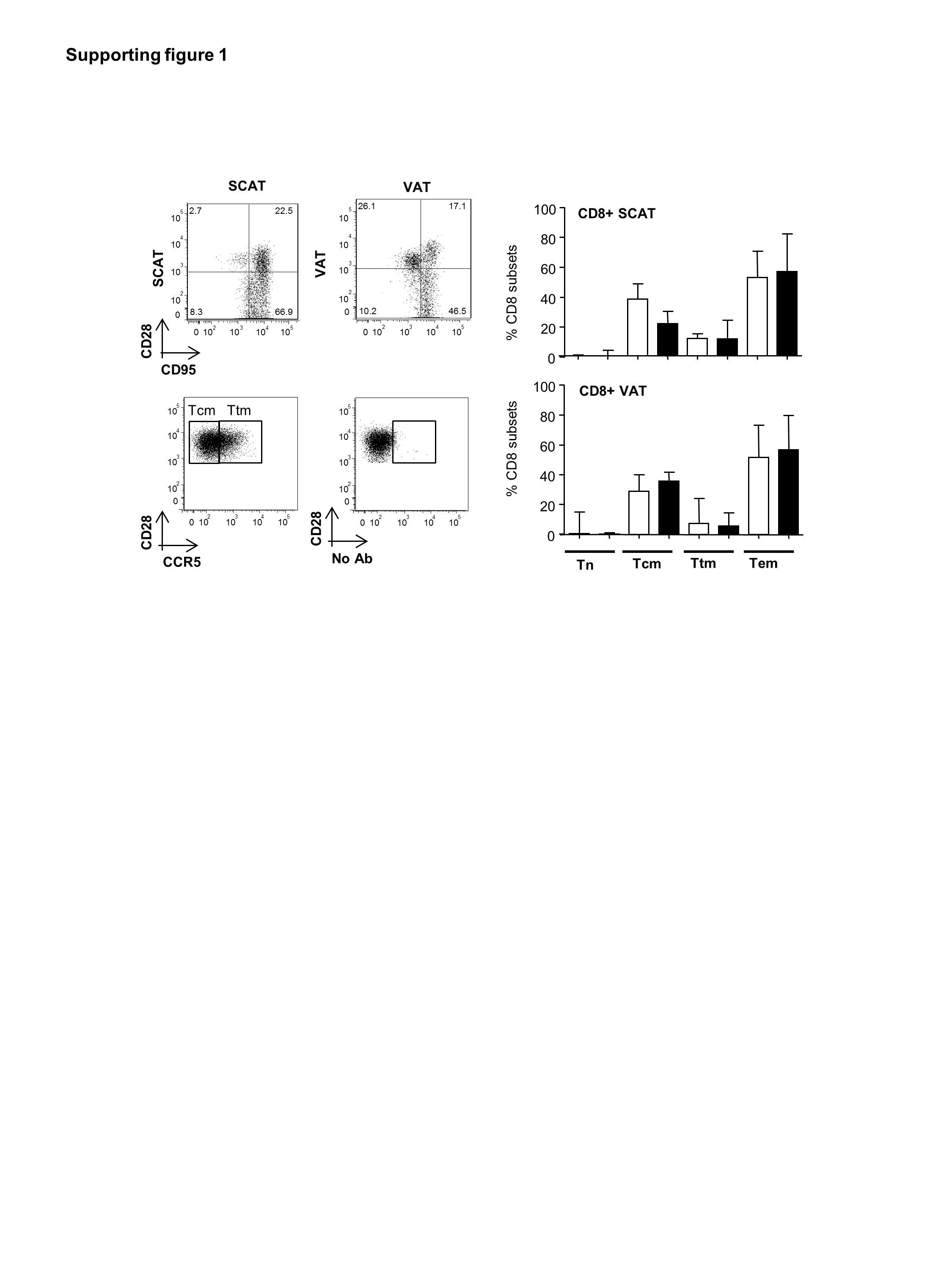

Supplement: S1 Fig — Representative dot plots showing the gating strategies used to define Tn, Tcm, Ttm and Tem subsets among CD8 T cells (based on CD28, CD95 and CCR5 staining) in SCAT and VAT. The right-hand panels show the distribution of CD8+ T cells among the different subsets in non-infected animals (n = 5, open column) and SIV-infected animals (n = 7, filled column). Data are quoted as the median [interquartile range]. A Mann-Whitney non-parametric test was used. (TIF) [file ppat.1005153.s001.tif]

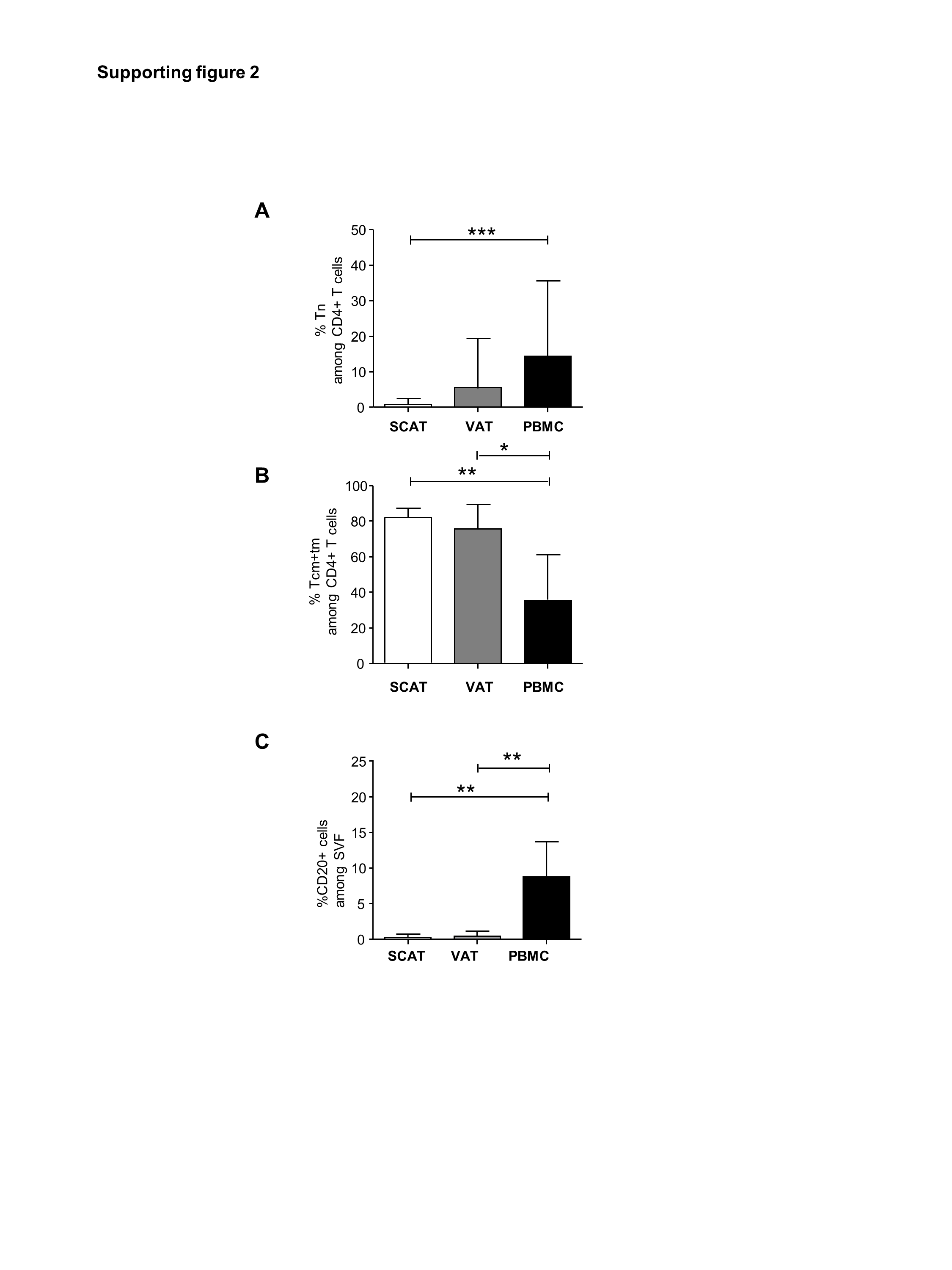

Supplement: S2 Fig — (A) The percentages of Tn CD4+ T cells (CD95+CD28int) in SCAT, VAT and PBMCs from pooled infected animals (n = 8) animals and non-infected animals (n = 8). (B) The percentage of Tm CD4+ T cells (CD95+CD28+CCR5+/-) in SCAT, VAT and PBMCs from infected and non-infected animals. (C) The percentage of CD20-expressing cells among the CD45+ fraction in SCAT, VAT and PBMCs from pooled infected animals (n = 5) and non-infected animals (n = 7). Datasets from SIV-infected animals (n = 7) and non-infected animals (n = 5–7) were pooled, since there was no apparent difference between the two groups. (TIF) [file ppat.1005153.s002.TIF]

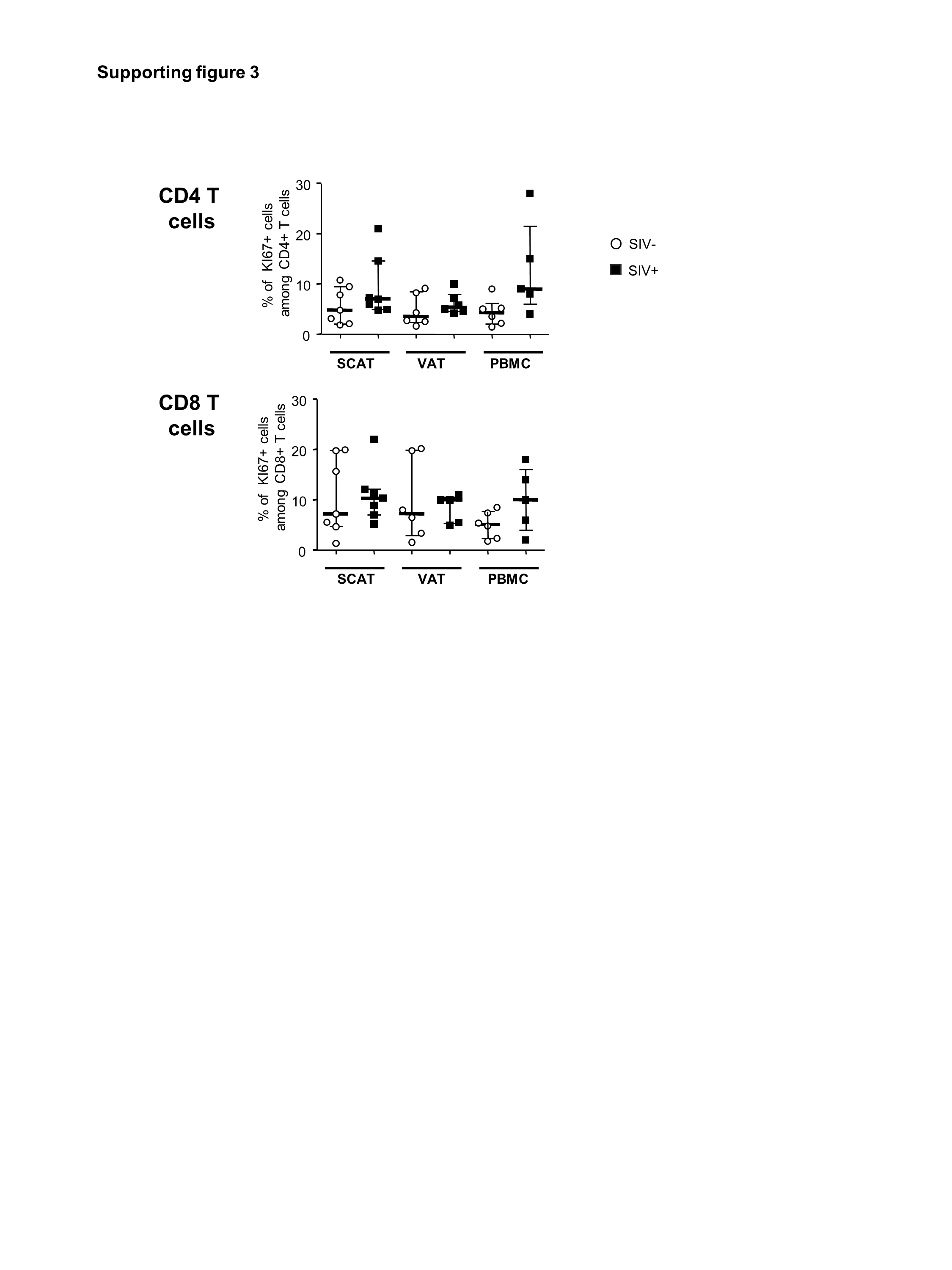

Supplement: S3 Fig — The percentage of Ki67-expressing cells among CD4+ and CD8+ T cells recovered from SCAT and/or VAT from 7 infected animals (filled symbols) and 7 non-infected animals (open symbols). Values from peripheral blood T cells are shown when available (n = 5–6). (TIF) [file ppat.1005153.s003.TIF]

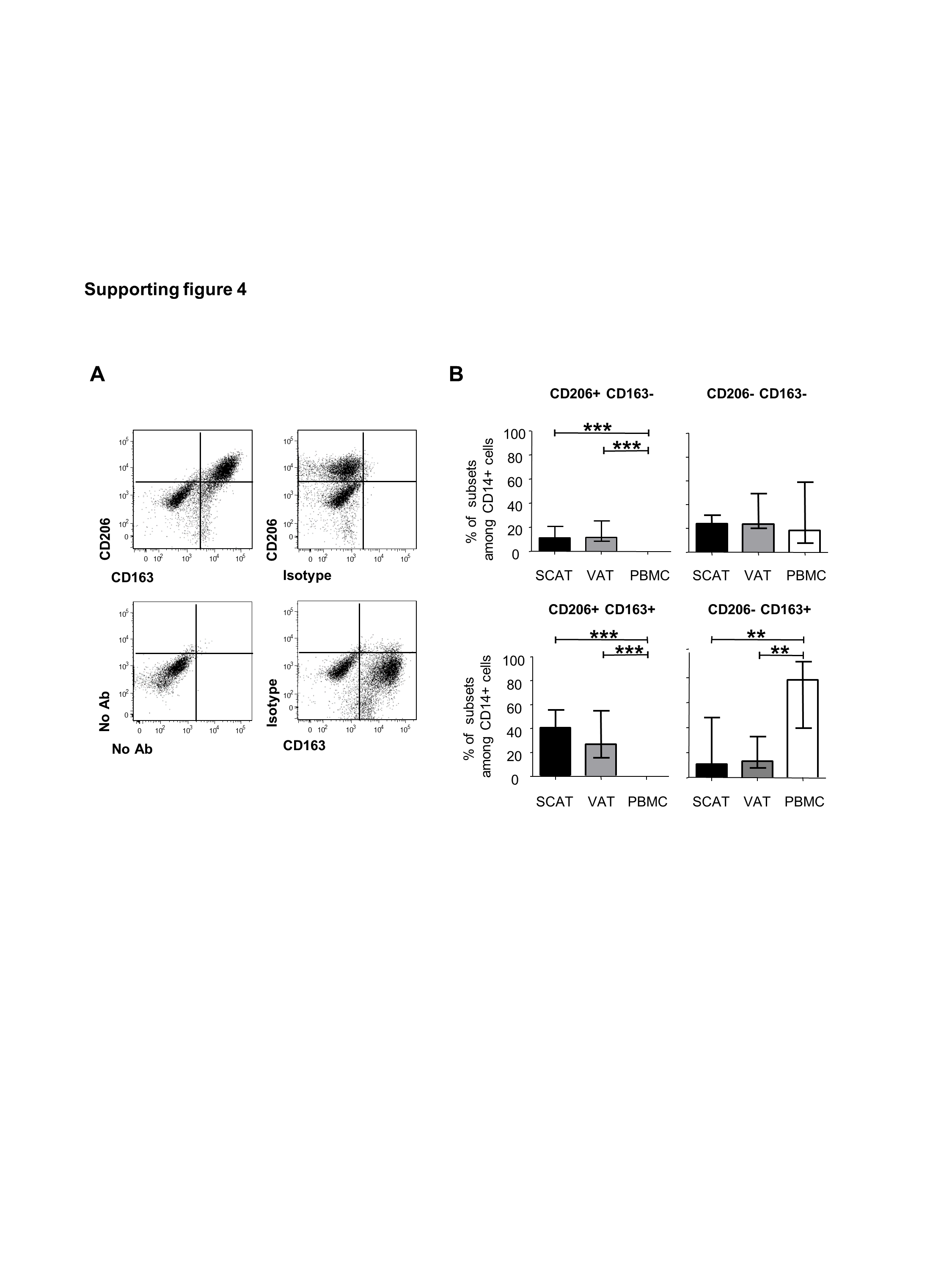

Supplement: S4 Fig — (A) Dot plots showing the co-expression of CD206 and CD163 on CD14-expressing cells. Due to high levels of auto-fluorescence, the gating strategy was defined using isotype controls for either anti-CD163 or anti-CD206 antibodies and an unstained approach for the CD163 and CD206 fractions. (B) Graphs showing the percentage of each fraction in CD14-expressing cells recovered from SCAT, VAT and PBMCs from 8 non-infected animals. Similar observations were made in SIV-infected animals. Data are quoted as the median [interquartile range]. Significant differences in a Mann-Whitney non-parametric test are shown as * p<0.05; ** p<0.01; *** p<0.001. (TIF) [file ppat.1005153.s004.TIF]

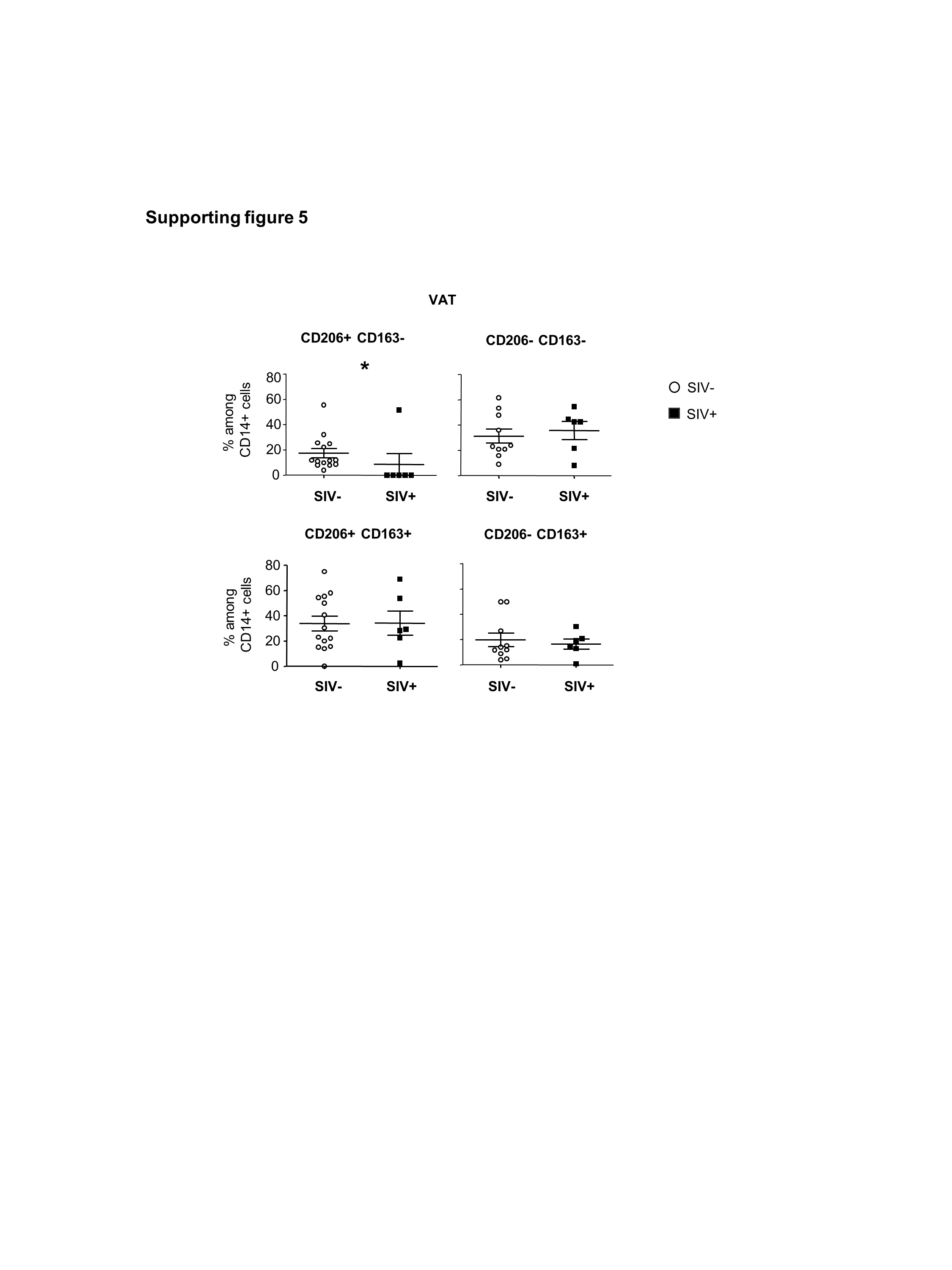

Supplement: S5 Fig — Expression of CD206 and CD163 on adipose-resident CD14-expressing cells recovered from VAT from non-infected animals (open circles, n = 8) and SIV-infected animals (filled squares, n = 6). Gating strategies are shown in S4 Fig. Data are quoted as the median [interquartile range]. Significant differences in a Mann-Whitney non-parametric test are shown as * p<0.05. (TIF) [file ppat.1005153.s005.TIF]

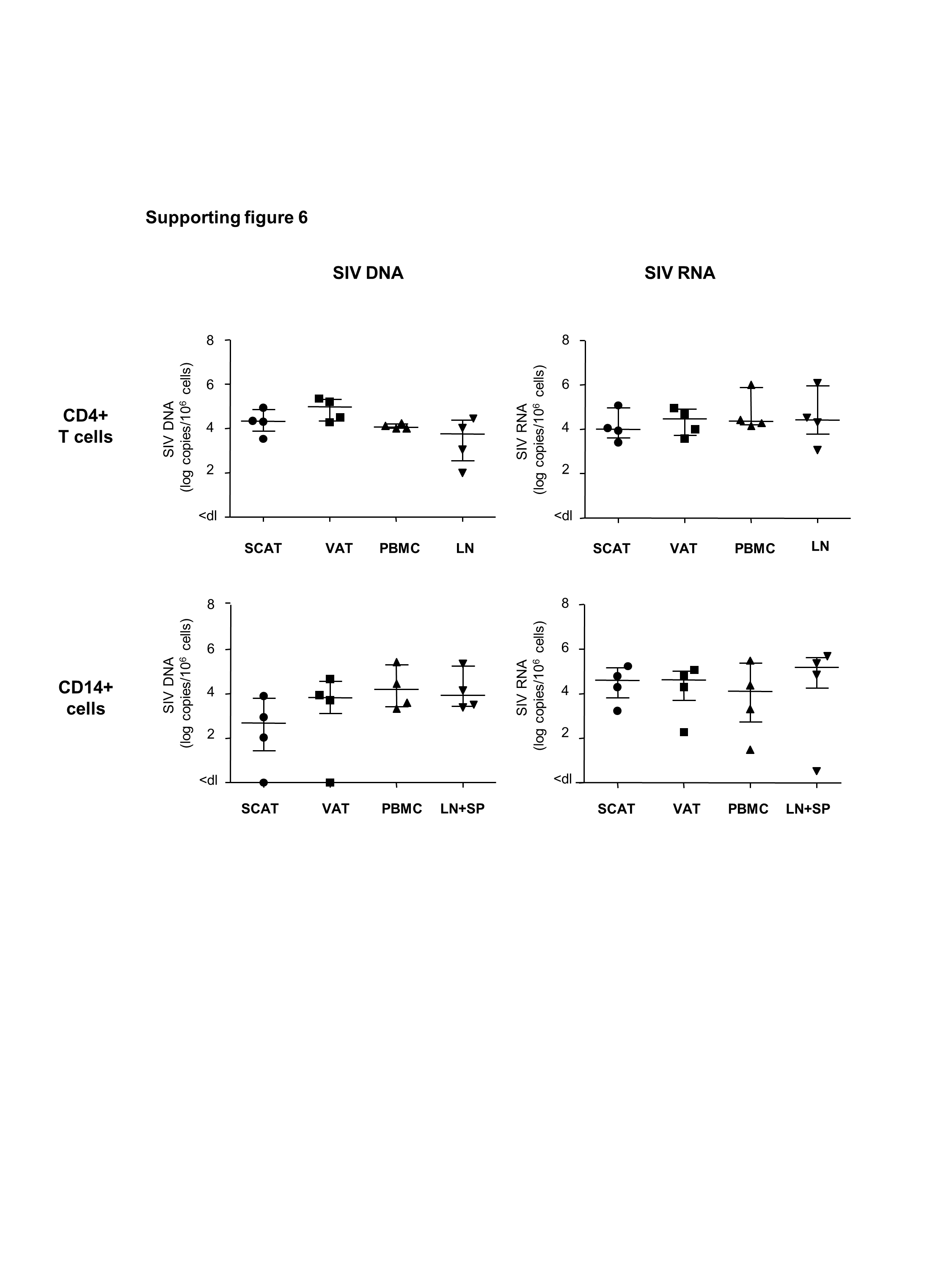

Supplement: S6 Fig — Comparison of SIV DNA and RNA levels in sorted CD4+ T cell and CD14+ cell fractions recovered from different organs (adipose tissue, PBMCs and lymph nodes) in four SIV-infected animals. SIV DNA and RNA assays were performed in duplicate and the results are expressed in log SIV DNA copies per million cells. Due to the low numbers of CD14+ cells recovered from lymph node, CD14+ cells were sorted from spleen for two animals. Data are quoted as the median [interquartile range]. A Mann-Whitney non-parametric test was used. (TIF) [file ppat.1005153.s006.TIF]

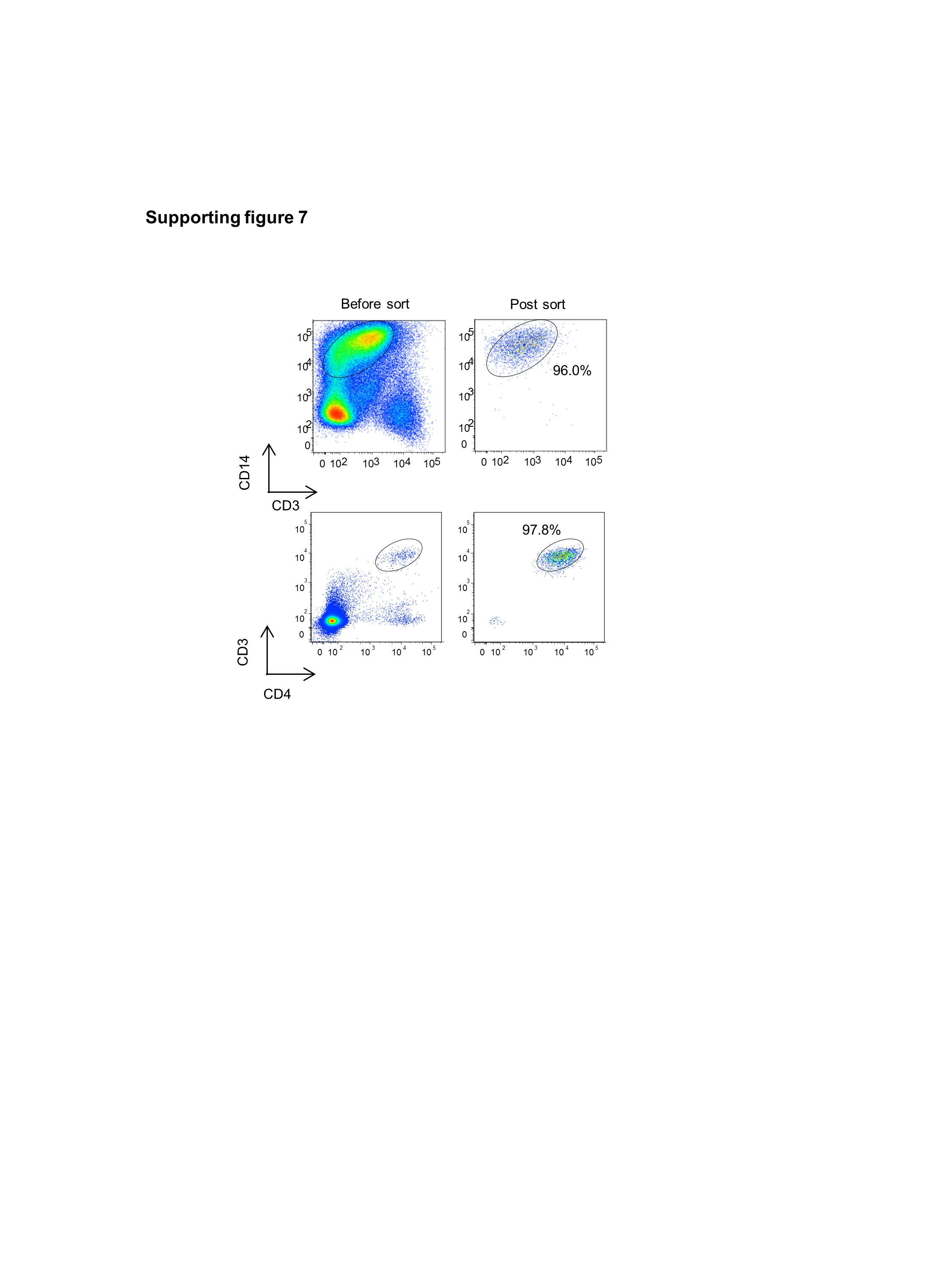

Supplement: S7 Fig — A representative sorting strategy used for CD14+ cells and CD4+ T cells. Dot plots of cells from a non-infected animal before and after sorting are shown. In both NHPs and humans, sorting purity was consistently over 95% (CD14+ cells: 97.2% [95.1–98.7], CD4+ T cells: 96.9% [96.1–99.1]). (TIF) [file ppat.1005153.s007.TIF]
